# Trimethoprim-sulfamethoxazole Versus Azithromycin for the Treatment of Undifferentiated Febrile Illness in Nepal: A Double-blind, Randomized, Placebo-controlled Trial

**DOI:** 10.1093/cid/ciaa1489

**Published:** 2020-09-29

**Authors:** Abhishek Giri, Abhilasha Karkey, Sabina Dangol, Amit Arjyal, Sunil Pokharel, Samita Rijal, Damodar Gajurel, Rabi Sharma, Kamal Lamsal, Pradip Shrestha, Gayatri Prajapati, Saruna Pathak, Sita Ram Shrestha, Raj Kumar K.C, Sujata Pandey, Abishkar Thapa, Nistha Shrestha, Raj Kumar Thapa, Buddhi Poudyal, Dung Nguyen Thi Phuong, Stephen Baker, Evelyne Kestelyn, Ronald Geskus, Guy Thwaites, Buddha Basnyat

**Affiliations:** 1 Oxford University Clinical Research Unit, Patan Academy of Health Sciences, Lalitpur, Nepal; 2 Patan Academy of Health Sciences, Lalitpur, Nepal; 3 Civil Service Hospital, Minbhawan Kathmandu, Nepal; 4 Oxford University Clinical Research Unit, Ho Chi Minh City, Vietnam; 5 University of Cambridge, Cambridge, United Kingdom; 6 Centre for Tropical Medicine and Global Health, University of Oxford, Oxford, United Kingdom

**Keywords:** typhoid fever, South Asia, health economics

## Abstract

**Background:**

Azithromycin and trimethoprim-sulfamethoxazole (SXT) are widely used to treat undifferentiated febrile illness (UFI). We hypothesized that azithromycin is superior to SXT for UFI treatment, but the drugs are noninferior to each other for culture-confirmed enteric fever treatment.

**Methods:**

We conducted a double-blind, randomized, placebo-controlled trial of azithromycin (20 mg/kg/day) or SXT (trimethoprim 10 mg/kg/day plus sulfamethoxazole 50 mg/kg/day) orally for 7 days for UFI treatment in Nepal. We enrolled patients >2 years and <65 years of age presenting to 2 Kathmandu hospitals with temperature ≥38.0°C for ≥4 days without localizing signs. The primary endpoint was fever clearance time (FCT); secondary endpoints were treatment failure and adverse events.

**Results:**

From June 2016 to May 2019, we randomized 326 participants (163 in each arm); 87 (26.7%) had blood culture–confirmed enteric fever. In all participants, the median FCT was 2.7 days (95% confidence interval [CI], 2.6–3.3 days) in the SXT arm and 2.1 days (95% CI, 1.6–3.2 days) in the azithromycin arm (hazard ratio [HR], 1.25 [95% CI, .99–1.58]; *P* = .059). The HR of treatment failures by 28 days between azithromycin and SXT was 0.62 (95% CI, .37–1.05; *P* = .073). Planned subgroup analysis showed that azithromycin resulted in faster FCT in those with sterile blood cultures and fewer relapses in culture-confirmed enteric fever. Nausea, vomiting, constipation, and headache were more common in the SXT arm.

**Conclusions:**

Despite similar FCT and treatment failure in the 2 arms, significantly fewer complications and relapses make azithromycin a better choice for empirical treatment of UFI in Nepal.

**Clinical Trials Registration:**

NCT02773407.

## Teaser

We compared azithromycin with trimethoprim-sulfamethoxazole for 7 days in the treatment of undifferentiated febrile illness in Nepal. Despite similar fever clearance time and treatment failure in the 2 arms, significantly fewer complications and relapses were noted in the azithromycin arm.

Fever without localizing signs of infection, otherwise known as undifferentiated febrile illness (UFI), is a common cause of morbidity and mortality in low- and middle-income countries [1–3]. The causes of UFI vary by geographic region. In South Asia, malaria, dengue, typhus, and *Salmonella* Typhi and paratyphoid fever are all common causes of UFI [1, 2]. Enteric fever is the commonest bacterial bloodstream infection in South Asia with an incidence of about 500 per 100 000 [4–6]. Widely available rapid diagnostic tests can help diagnose malaria and dengue, but distinguishing enteric fever from scrub or murine typhus, or other less common causes, is difficult. This diagnostic challenge is compounded by rapidly rising drug resistance among *S.* Typhi and paratyphoid fever, especially against the fluoroquinolones [4, 7], which makes the selection of appropriate antimicrobial treatment difficult. These uncertainties threaten treatment outcomes and drive escalating and poorly directed antimicrobial use.

The commonest causes of UFI in Nepal are *S.* Typhi and paratyphoid fever, murine and scrub typhus, and leptospirosis [8, 9]. Pooled data from 2092 patients with UFI enrolled in 4 previous clinical trials conducted in Kathmandu showed that 885 (41%) had either *S.* Typhi or paratyphoid fever [10]. Serological testing of a subset of patients recruited in these studies showed evidence for murine typhus in 17% (n = 21/125), with the spotted fever group rickettsioses, Q fever, hantavirus infection, brucellosis, and dengue as additional causes of UFI [9].

Many patients with UFI in Nepal and other parts of South Asia are treated empirically [11] for enteric fever. In Nepal and the wider region, the selected antimicrobials depend upon availability and cost, but trimethoprim-sulfamethoxazole (SXT) and azithromycin are very commonly prescribed antibiotics [12, 13]. Currently, both *S.* Typhi and paratyphoid fever are highly susceptible to both SXT and azithromycin in Nepal [10].

SXT was commonly used in the past for enteric fever treatment [14–18], but the emergence of multidrug-resistant *S.* Typhi, which was SXT resistant, 2 decades ago reduced its use. However, in the last few years SXT resistance has largely disappeared and nearly all *S.* Typhi and *Salmonella* Paratyphi A isolates from Nepal and the nearby region are now susceptible [7, 19–21]. There are no recent clinical trials, but its effectiveness against enteric fever is supported by a recent case report [22] and observed low SXT minimum inhibitory concentrations (MICs) for *S.* Typhi and *S.* Paratyphi A [20, 23].

In 2019, the Indian Council of Medical Research antimicrobial guidelines recommended SXT or azithromycin as the initial, preferred treatment for suspected enteric fever [24]. Azithromycin is very effective for enteric fever, with minimal resistance currently reported [25]. It may also have some activity against scrub and murine typhus. There have been no trials performed for the treatment of UFI in settings with endemic fluoroquinolone-resistant *S.* Typhi. There have been several published studies that used azithromycin [26–28] or SXT for the treatment of enteric fever, but there have been no head-to-head randomized comparisons of the 2 drugs for UFI. In addition, many of the previous SXT studies had small sample sizes and were conducted in the 1970s and 1980s [14–18]. Therefore, we conducted a randomized, double-blind comparison of azithromycin vs SXT for the treatment of UFI in Nepal. We hypothesized [29] that azithromycin would be superior to SXT for the treatment of patients with UFI and sterile blood cultures but that the 2 drugs would be noninferior to one another for the treatment of blood culture–confirmed enteric fever.

## METHODS

### Study Design and Participants

We conducted a parallel-group, double-blind, randomized controlled trial of SXT vs azithromycin for the treatment of UFI in Nepal at Patan Hospital and Civil Services Hospital in the Kathmandu Valley, Nepal. [29] The study protocol was reviewed and approved by the Ethics Committee of the Nepal Health Research Council and the Oxford Tropical Research Ethics Committee, United Kingdom.

We screened patients between >2 years and <65 years of age who presented at the emergency room and outpatient clinics of Patan Hospital and Civil Service Hospital, who had a temperature of ≥38.0°C and a documented or self-reported history of fever for ≥4 days and <14 days, without a localizing focus of infection. Patients were excluded if they were pregnant; had signs of severe infection (eg, obtunded, in shock, had severe jaundice, or active gastrointestinal bleeding) that required intravenous antibiotics or hospital admission; had a history of hypersensitivity to either of the trial drugs; were already on antimicrobials and responding; or if the study physician considered either drug was contraindicated for any reason.

Written informed consent to participate in the study was obtained from all patients ≥18 years of age. For patients aged 12–17 years, written informed consent was obtained from a legal guardian in addition to assent from the participant. Written informed consent was obtained from legal guardians for patients <12 years of age.

### Randomization and Blinding

All enrolled patients were randomly assigned (1:1) to either SXT or azithromycin according to a computer-generated randomization list, with randomization in variable block sizes of 4 and 6 without stratification. The randomization list specified the assignment of each unique study number to the respective randomized treatment arm.

At enrollment, the study staff explained how the drugs should be taken by the patient (Supplementary Table 1). Treatment allocation was concealed from the patient, investigators, study physicians, nurses, and other study staff throughout the study.

### Procedures

Patients were randomized to either 1 of the 2 treatment groups: Group A was administered azithromycin tablets 20 mg/kg/day as a single daily dose orally for 7 days (maximum dose 1000 mg/day); and group B was administered SXT tablets (trimethoprim 10 mg/kg + sulfamethoxazole 50 mg/kg) in 2 divided doses daily orally for 7 days (maximum 3000 mg/day). The tablets were manufactured by Lomus Pharmaceuticals Nepal as follows: SXT tablets of 1200 mg, 600 mg, 300 mg, and 150 mg; azithromycin tablets of 800 mg, 400 mg, 200 mg, and 100 mg; and placebo tablets in 4 different sizes. The placebo tablets were identical to the active drug. The content of the placebo and the drug doses adjusted according to the weight of individual patients is given in Supplementary Table 2 and Supplementary Table 3.

The follow-up intervals and the assessment schedule are given in Supplementary Table 4. The blood culture and antibiotic susceptibility were done similarly as described in our previous trial [7].

### Outcomes

The primary endpoint was fever clearance time (FCT): that is, the time from the first dose of the study drug until a temperature of ≤37 .5°C was recorded for at least 48 hours.

The secondary endpoint was treatment failure, defined as the occurrence of at least 1 of the following events: FCT >7 days (168 hours) after treatment initiation; clinical failure and requirement for rescue treatment as judged by the study physician and the attending physician; blood culture positive for *S.* Typhi or paratyphoid fever on day 7 of treatment (microbiological failure); culture-confirmed or syndromic enteric fever relapse within 28 days of initiation of treatment; development of any complication (eg, clinically significant bleeding, decline in Glasgow Coma Scale, perforation of the gastrointestinal tract, and need for hospital admission within 28 days after the initiation of treatment). The time to treatment failure was defined as the time from the first dose of treatment until the date of the earliest failure event. Adverse events were also secondary endpoints and defined as grade 3/4 adverse events, serious adverse events, adverse events of any grade leading to modification of study drug dose, or interruption/early discontinuation.

Patients who met the criteria for treatment failure were given intravenous ceftriaxone 60 mg/kg once daily (maximum dose 2 g/day) for 7 days if they had culture-confirmed enteric fever. Those with sterile blood cultures were treated with intravenous ceftriaxone 60 mg/kg once daily (maximum dose 2 g/day) and oral doxycycline (4 mg/kg/day) in 2 divided doses (maximum 200 mg/day) for 7 days.

### Statistical Analysis

Analyses were prespecified in a statistical analysis plan before unblinding of the treatment allocation. Based on data from our previous trial [7], we assumed a Weibull distribution for the FCT in each arm and a median FCT in the azithromycin arm of 1.12 days in those with culture-negative UFI and 2.78 days in those with culture-positive UFI. The shape parameter of the Weibull distribution was assumed to be 0.75 for culture-negative patients and 1.5 in culture-positive patients, which was a conservative estimate compared to the observed FCT distributions in the previous 3 trials we conducted [23, 30, 31]. We assumed that SXT would be associated with a 2-fold slower FCT in culture-negative patients and the same FCT as azithromycin in culture-positive patients. Finally, we assumed that the proportion of patients with culture-confirmed enteric fever would be 33%–50%.

Based on these assumptions and an assumed twice-daily temperature monitoring leading to an interval-censored FCT, the power for various samples sizes was estimated based on simulations (Supplementary Table 5). The target sample size chosen was 330 participants (165 per study group), which included an allowance of 10% loss to follow-up and providing ≥80% power for the overall comparison with a 2-sided 5% significance level.

The primary analysis population was by intention-to-treat, with prespecified subgroup analyses for both primary and secondary outcomes in those with or without blood culture–confirmed enteric fever (except for relapse, the subgroup analyses for the individual components of treatment failure were not part of the statistical analysis plan). The primary endpoint FCT was compared between the 2 groups based on a Weibull accelerated failure time model with the treatment arm as the only covariate. The distributions of the FCT over time in each treatment arm were further visualized using the nonparametric maximum likelihood estimator for interval-censored data. With respect to treatment failure and its individual components, we compared the 2 groups with a Cox regression model with treatment as the only covariate. We used Firth penalized likelihood in case the number of events in one of the arms was zero or 1. We computed the distribution of time to treatment failure via Kaplan-Meier curves and compared the absolute risk of treatment failure until day 28. Comparisons of the number of patients with each adverse event between the 2 arms were done with Fisher exact test. None of the *P* values were corrected for multiple comparisons. All analyses were done with R language for statistical computing version 3.6.2 software [32].

The safety of the trial was overseen by an independent data and safety monitoring board. The trial was registered at ClinicalTrials.gov (identifier NCT02773407).

## RESULTS

**Figure 1.**
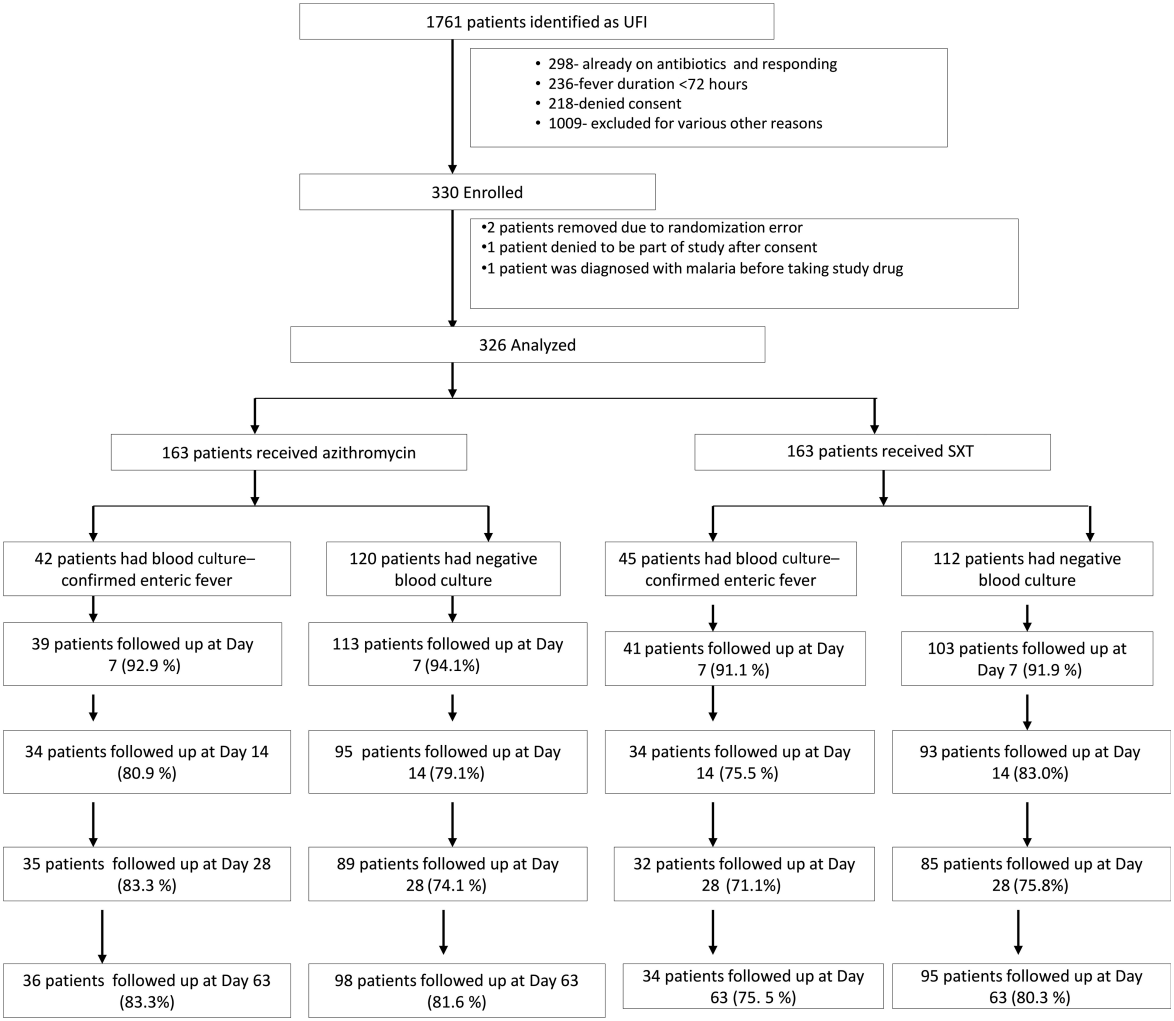
Trial participant flowchart. Abbreviations: SXT, trimethoprim-sulfamethoxazole; UFI, undifferentiated febrile illness.

Between June 2016 and May 2019, 326 patients were randomized to either arm (Figure 1). The baseline characteristics were well-balanced between the groups (Table 1).

**Table 1.** Baseline Characteristics of the Trial Participants According to Treatment Group (Intention-to-Treat Population)

Characteristic	SXT (n = 163)	Azithromycin (n = 163)
Age, y		
Median (1st–3rd quartile)	22.0 (16.2–29.0)	21.0 (16.0–28.8)
<14	22 (13.4)	28 (17.1)
≥14	140 (85.8)	134 (82.2)
Sex		
Male	113 (69.3)	104 (63.8)
Female	60 (30.7)	59 (36.2)
Median days of illness (1st–3rd quartile)	5.0 (4.0–6.0)	5.0 (4.0–6.0)
Risk factors		
Drinking water source		
Tap water (piped supply)	69 (42.3)	70 (42.9)
Well	1 (0.6)	6 (3.7)
Tube well	3 (1.8)	4 (2.5)
Stone spout	5 (3.1)	4 (2.5)
Bottled water	58 (35.6)	55 (33.7)
Others	27 (16.6)	24 (14.7)
Treatment of drinking water		
Untreated	72 (44.2)	76 (46.6)
Filtered	52 (31.9)	57 (35.0)
Boiled	26 (16.0)	22 (13.5)
Boiled and filtered	8 (4.9)	3 (1.8)
Chlorinated	4 (2.5)	2 (1.2)
Others	1 (0.6)	3 (1.8)
Food taken outside of usual place in last 3 wk		
Restaurants/small hotels	50 (30.7)	51 (31.3)
Street vendors	10 (6.1)	10 (6.1)
Party	10 (6.1)	7 (4.3)
None other than the usual place	87 (53.4)	90 (55.2)
Others	6 (3.7)	5 (3.1)
Symptoms		
Median temperature at presentation, °C (1st–3rd quartile)	38.2 (37.3–38.7)	38.3 (37.6–38.8)
Fever	163 (100)	163 (100)
Headache	136 (83.4)	143 (87.7)
Anorexia	130 (79.8)	126 (77.3)
Nausea	78 (47.9)	70 (42.9)
Vomiting	50 (30.7)	49 (30.1)
Diarrhea	43 (26.4)	42 (25.8)
Constipation	19 (11.7)	27 (16.6)
Abdominal pain	45 (27.6)	50 (30.7)
Black stool	10 (6.1)	6 (3.7)
Cough	76 (46.6)	83 (50.9)
Chest pain	17 (10.4)	19 (11.7)
Throat discomfort	26 (16.0)	24 (14.7)
Weakness	121 (74.2)	114 (69.9)
Acute gastroenteritis	19 (11.8)	26 (16.0)
Prior antibiotics	49 (32.9)	53 (36.1)
Laboratory values, median (1st–3rd quartile)		
Hematocrit, %	41.0 (39.0–44.9)	41.0 (38.0–45.0)
WBC count, ×10^9^ cells/L	5.7 (4.8–7.2)	6.3 (6.0–7.4)
Platelets, ×10^9^ cells/L	186 (152–236)	204 (164–252)
Neutrophils, %	68.0 (62.0–76.0)	70.0 (63.0–78.0)
Lymphocytes, %	30.0 (24.0–36.0)	28.0 (21.2–35.0)
AST, U/L	52.5 (36.0–80.0)	54.0 (35.0–74.0)
ALT, U/L	50.5 (36.2–85.8)	48.0 (34.0–75.6)
Blood culture and sensitivity		
Contaminants	6 (3.7)	1 (0.6)
No growth	112 (68.7)	120 (73.6)
*Salmonella* Typhi	41 (25.2)	39 (23.9)
*Salmonella* Paratyphi A	4 (2.5)	3 (1.8)
Stool culture and sensitivity		
*Salmonella* Typhi	4 (2.9)	2 (1.5)

Data are presented as no. (%) unless otherwise indicated.

Abbreviations: ALT, serum alanine aminotransferase; AST, serum aspartate aminotransferase; SXT, trimethoprim-sulfamethoxazole; WBC, white blood cell.

**Figure 2.**
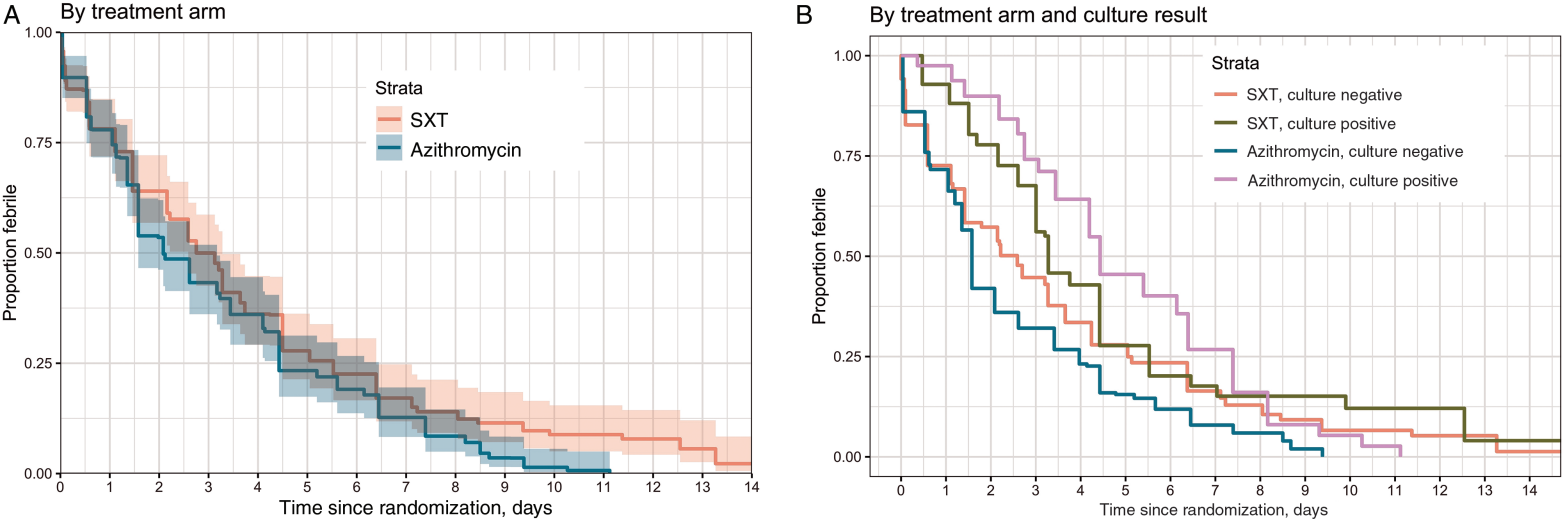
Estimated time to fever clearance by treatment arm in the intention-to-treat population (*A*) and the subgroup of culture-confirmed and culture-negative patients (*B*). Abbreviation: SXT, trimethoprim-sulfamethoxazole.

**Figure 3.**
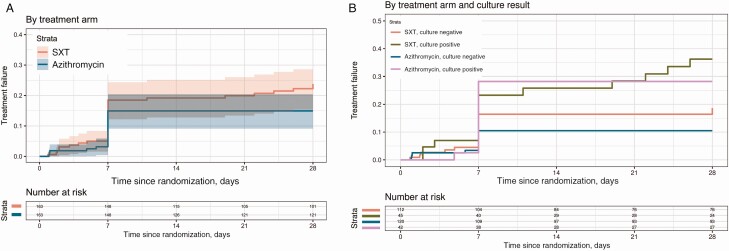
Kaplan-Meier curves for time to treatment failure in 2 treatment groups in the intention-to-treat population (*A*) and the subgroups of culture-positive and culture-negative patients (*B*). Abbreviation: SXT, trimethoprim-sulfamethoxazole.

The median FCT was 2.7 days (95% confidence interval [CI], 2.6–3.3 days) in the SXT arm and 2.1 days (95% CI, 1.6–3.2 days) in the azithromycin arm. The hazard ratio (HR) for the treatment effect (azithromycin vs SXT) was 1.25 (95% CI, .99–1.58) (*P* = .059; Figure 2A). The probability of the secondary and composite endpoint of treatment failure at 28 days was 0.15 (95% CI, .09–.20) in the azithromycin arm vs 0.24 (95% CI, .17–.30) in the SXT arm (HR, 0.62 [95% CI, .32–1.05]; *P* = .073) (Table 2 and Figure 3A). This difference was driven primarily by lower numbers of syndromic or culture-confirmed relapses (6 SXT; 0 azithromycin; HR, 0.07 [95% CI, .00–.56]; *P* = .008) and lower numbers of enteric fever–related complications (8 SXT, 1 azithromycin; HR, 0.17 [95% CI, .02–.97]; *P* = .011) within 28 days of treatment initiation in the azithromycin arm (Table 3).

**Table 2.** Primary and Secondary Endpoints in the Intention-to-Treat Population and Subgroups of Blood Culture–Positive and Culture-negative Patients

Endpoint	SXT	Azithromycin	Comparison, HR (95% CI)	*P* Value
Fever clearance time, d, median (95% CI)				
Overall ITT	2.7 (2.6–3.3)	2.1 (1.6–3.2)	1.25 (.99–1.58)	.059
Culture positive	3.9 (3.0–4.4)	4.4 (4.2–6.4)	0.95 (.60–1.48)	.81
Culture negative	2.6 (1.4–3.3)	1.6 (1.3–2.1)	1.37 (1.04–1.80)	.025
Treatment failure within 28 days				
Overall ITT	36/163	23/163	0.62 (.37–1.05)	.073
Probability within 28 days, % (95% CI)	24 (17–30)	15 (9–20)	…	
Culture positive	14 (37)	11 (37)	0.78 (.35–1.72)	.537
Probability within 28 days, % (95% CI)	39 (21–53)	30 (13–43)	…	
Culture negative	20 (112)	12 (120)	0.56 (.27–1.14)	.11
Probability within 28 days, % (95% CI)	19 (11–26)	11 (5–16)	…	

Abbreviations: CI, confidence interval; HR, hazard ratio (based on Weibull regression mode for fever clearance time and Cox regression for treatment failure); ITT, intention-to-treat; SXT, trimethoprim-sulfamethoxazole.

**Table 3.** Components of Treatment Failure in the Intention-to-Treat Population and Subgroups of Blood Culture–Positive and Culture-negative Patients

Component	SXT	Azithromycin	Comparison (95% CI)	*P* Value
Fever failure at day 7				
Overall ITT	22/134	18/135	RR, 1.20 (.67–2.14)	.54
Probability, % (95% CI)	14 (9–20)	12 (8–18)	…	
Culture positive	7 /36	10/29	RR, −0.63 (.27–1.51)	.30
Probability, % (95% CI)	16 (8–30)	26 (14–41)	…	
Culture negative	14/95	8/105	RR, 1.81 (.79–4.15)	.16
Probability, % (95% CI)	13 (8–21)	7 (4–14)	…	
Clinical failure within day 7				
Overall ITT	8/163	5/163	HR, 0.63 (.20–1.92)	.41
Failure probability within day 7, % (95% CI)	5.0 (1.6–8.3)	3.2 (.4–5.9)	…	
Culture positive	3/45	1/42	HR, 0.44 (.04–2.67)	.38
Failure probability within day 7, % (95% CI)	7 (0–14)	2.6 (0–7.4)	…	
Culture negative	5/118	4/121	HR, 0.77 (.21–2.85)	.69
Failure probability within day 7, % (95% CI)	0.04 (.01–.08)	0.03 (.00–.07)	…	
Microbiological failure	0	1		
Syndromic or culture confirmed relapse till day 28				
Overall ITT	6/126	0/130	HR, 0.007 (.00–.56)	.008
Probability within 28 days, % (95% CI)	5.6 (1.1–9.8)	0	…	
Culture positive	5/33	0/28	HR, 0.09 (.00–.81)	.028
Probability within 28 days, % (95% CI)	17 (2–29)	0	…	
Culture negative	1/90	0/101	HR, 0.27 (.00–5.11)	.39
Probability within 28 days, % (95% CI)	1.3 (.0–3.8)	0	…	
Complications till day 28				
Overall ITT	8/163	1/163	HR, 0.17 (.02–.97)	.011
Probability within 28 days, % (95% CI)	5.4 (1.7–8.9)	0.6 (.0–1.8)	…	
Culture positive	4/45	0/42	HR, 0.11 (.00–1.05)	.056
Probability within 28 days, % (95% CI)	10 (0–18)	0	…	
Culture negative	4/112	1/120	HR, 0.31 (.03–1.68)	
Probability within 28 days, % (95% CI)	3.9 (.1–7.5)	0.8 (.0–2.4)	…	.18
Rescue treatment	23	11		
Overall ITT	23/163	11/163	…	
Culture positive	10/45	5/42	…	
Culture negative	13/112	6/120	…	

Abbreviations: CI, confidence interval; HR, hazard ratio (based on Cox regression); ITT, intention-to-treat population; RR, relative risk; SXT, trimethoprim-sulfamethoxazole.

There was heterogeneity in the primary outcome between the prespecified subgroups of those with *S.* Typhi or paratyphoid fever cultured from blood (culture positive) and those with sterile blood cultures (culture negative) at baseline (*P* for interaction = .088). The median FCT in the culture-positive group was 3.3 days (95% CI, 3.0–4.4 days) in the SXT arm and 4.4 days (95% CI, 4.2–6.4 days) in the azithromycin arm (HR, 0.95 [95% CI, .60–1.48]; *P* = .808). However, in the culture-negative participants, the median FCT was 2.6 days (95% CI, 1.4–3.3 days) in the SXT arm and 1.6 days (95% CI, 1.3–2.1 days) in the azithromycin arm (HR, 1.37 [95% CI, 1.04–1.80]; *P* = .025) (Figure 2B). In the model with culture and the interaction with treatment added (Figure 2B), the overall *P* value for the treatment effect was .031. The *P* value for the interaction between culture and treatment was .088; the overall *P* value for the culture effect was <.001.We did not observe heterogeneity of the treatment effect on the composite secondary endpoint of treatment failure by 28 days between the 2 prespecified subgroups (*P* for effect modification = .51) (Table 2).

There was no relapse in either of the subgroups in the azithromycin arm and only 1 complication in the culture-negative group in the azithromycin arm.The HR of treatment effect for relapse in the culture-postive group within 28 days was 0.09 (95% CI, .00–.81; *P* = .028). The HR of treatment effect for complications in the culture-positive group within 28 days was 0.11 (95% CI, .00–1.05; *P* = .056) (Table 3). In the model with culture and the interaction with treatment added, the overall *P* value for the treatment effect was .21 (Figure 3B). The *P* value for the interaction between culture and treatment was 0.51; the overall *P* value for the culture effect was .007. The heterogenity tests for interval censored fever clearance time for other subgroups (age, sex, MIC) besides the prespecified subgroups are shown in (Supplementary Table 6).

There were 4 culture-confirmed relapses and 2 syndromic relapses within 28 days of treatment initiation in the SXT arm (Table 3). Eleven patients had to be admitted to hospital for grade 3 or 4 adverse events, high-grade or persistent fever, or on 1 occasion for administration of intravenous rescue treatment. Nine were in the SXT arm and 2 were in the azithromycin arm [Table 4]. There were 34 rescue treatments given during the study; 23 were in the SXT arm and 11 in the azithromycin arm. Persistent fever constituted the most common cause for rescue treatment. Twenty-one of 34 (61.8%) rescue treatments were given for persistent fever at day 7. One culture-positive patient had *S.* Typhi resistant to SXT, chloramphenicol, and amoxicillin in vitro but nevertheless responded well to SXT. All of the other isolates were susceptible to SXT and azithromycin. The MICs for different drugs among the isolates are given in (Supplementary Table 7).

**Table 4.** Adverse Events in the Study Participants According to Treatment Group

	SXT		Azithromycin		
Adverse Event	No. of Patients (%)	No. of AEs	No. of Patients (%)	No. of AEs	Comparison *P* Value
Grade 4					
Dyspnea	1 (0.61)	1	1 (0.61)	1	1
Grade 3					
Any AE	4 (2.45)	4	0	0	.123
Cough	1 (0.61)	1	0	0	1
Hepatobillary jaundice	1 (0.61)	1	0	0	1
Maculopapular rash	1 (0.61)	1	0	0	1
Grade 1/grade 2					
Any AE	135 (82.82)	448	13 (81.6)	381	.885
Vomiting	34 (20.86)	38	16 (9.82)	17	.008
Anorexia	31 (19.02)	32	24 (14.72)	24	.375
Black stool	3 (1.84)	3	5 (3.07)	5	.723
Chest pain	13 (7.98)	13	19 (11.66)	19	.352
Constipation	20 (12.27)	22	7 (4.29)	7	.014
Cough	31 (19.02)	34	36 (22.09)	39	.584
Dizziness	39 (23.93)	42	33 (20.25)	37	.505
Fever	3 (1.84)	5	1 (0.61)	1	.623
General weakness	42 (25.77)	45	37 (22.7)	40	.605
Headache	37 (22.7)	41	23 (14.11)	23	.062
Joint pain	14 (8.59)	14	19 (11.66)	19	.463
Loose stool	25 (15.34)	27	50 (30.67)	56	.001
Maculopapular rash	9 (5.52)	9	5 (3.07)	5	.414
Nausea	39 (23.93)	44	25 (15.34)	28	.069
Abdominal pain	34 (20.86)	39	30 (18.4)	32	.676

Abbreviations: AE, adverse event; SXT, trimethoprim-sulfamethoxazole.

## DISCUSSION

We compared azithromycin with SXT for 7 days in the treatment of UFI in Nepal. In all participants, azithromycin was associated with shorter FCT, fewer treatment failures, and fewer adverse events (with the exception of diarrhea, which was more common in the azithromycin arm), although the difference in FCT and treatment failure did not reach standard statistical significance (*P* = .059 and *P* = .073, respectively). However in the 2 prespecified trial subgroups, defined by positive or negative blood cultures for *S.* Typhi or paratyphoid fever, the drugs had different treatment outcomes. The FCT of both drugs was similar in those with positive cultures, but azithromycin was associated with significantly shorter FCT than SXT in culture-negative participants. In addition, although there were no differences in treatment failure in both the subgroups, there were significantly more relapses in the SXT arm in culture-positive participants.

Azithromycin and SXT are commonly used antibiotics in South Asia for the treatment of UFI before blood culture results are available. An analysis of pharmaceutical sales in India revealed that SXT and azithromycin are among the top 5 antibiotics sold [12]. The recent decrease in multidrug-resistant *S.* Typhi in the region, and the return of SXT-susceptible bacteria, has meant this inexpensive antibiotic might be potentially used in UFI treatment [19, 22]. Indeed, SXT is now recommended by some authorities as the preferred treatment [24] of suspected enteric fever and other UFIs. Therefore, our findings, which suggest that 7 days of SXT is inferior to azithromycin in the treatment of UFI and enteric fever, are important for clinicians and policy makers.

There has not been any recent study reported on SXT for enteric fever treatment. The 7 previous trials, published between 1972 and 1989 [14–16, 33, 34] (Supplementary Table 8) used lower doses of SXT (800–1600 mg/day; 15–25 mg/kg/day) than our trial (60 mg/kg/day to maximum 3000 mg/day). These trials employed variable endpoints, and only 3 followed up patients for relapse (Supplementary Table 8). However in the present study, despite the higher doses, there was an unacceptably high relapse rate (17%) in the SXT-treated patients with blood culture–confirmed enteric fever. This finding suggests that 7 days of SXT fails to kill all the bacteria and longer durations may be required to induce relapse-free cure. Historically, 14 days of SXT have been given for enteric fever treatment, although without support from randomized comparisons of treatment duration.

In the culture-negative participants, azithromycin cleared fever more rapidly (Figure 2B) than SXT. This rapid clearance may be due to the fact that murine typhus (and possibly scrub typhus) accounts for many UFIs, as our previous studies have shown [9]. Rapid clearance of fever within 48 hours with effective antibiotic treatment is usual in most cases of murine and scrub typhus treatment [34–36]. In addition, both murine [37] and scrub [38] typhus are known to be effectively treated with azithromycin. We do not know of any recent studies using cotrimoxazole in the treatment of rickettsial illnesses. Importantly, the lack of affordable, accurate rapid diagnostic tests for rickettsial diseases is a major hindrance for proper treatment.

The strengths of our trial include that it was placebo-controlled and pragmatic, enrolling participants from 2 hospitals in Kathmandu who were representative of a common and important clinical syndrome. We employed clinically relevant endpoints and followed participants for 63 days. It is also the first randomized controlled trial to investigate the use of SXT for the treatment of enteric fever since the 1980s, when antimicrobial use and bacterial and drug resistance epidemiology were very different from the present day.

The study has limitations. First, 7 days of SXT may have been too short, and may have accounted for the higher relapse rate in those with culture-confirmed enteric fever compared with azithromycin. Second, the cause of UFI was undefined in the majority of participants, which leaves uncertainty as to why SXT was associated with longer FCTs in the culture-negative group.

## CONCLUSIONS

These findings make azithromycin a better choice for empirical treatment of UFI in Nepal and other settings where enteric fever is common. However, understanding the local epidemiology, including resistance patterns, is critical for optimal clinical care [39]. The challenges diagnosing and treating UFI, especially when it is caused by *S.* Typhi, highlight the need for new point-of-care diagnostics and the value of the new typhoid conjugate vaccine, recently trialed in Nepal [40].

## Supplementary Data

Supplementary materials are available at *Clinical Infectious Diseases* online. Consisting of data provided by the authors to benefit the reader, the posted materials are not copyedited and are the sole responsibility of the authors, so questions or comments should be addressed to the corresponding author.

ciaa1489_suppl_Supplementary_Tables

ciaa1489_suppl_Supplementary_Material

## Notes

***Author contributions.*** B. B., A. K., A. A., S. P., S. B., G. T., and S. D. designed and conceived the study. All authors ran the study and contributed data. R. G., B. B., G. T., A. G., and A. A. did the data analysis. A. A., A. K., S. D., S. P., A. T., S. P., A. G., S. P., and R. K. did the experiments contributing to this study. B. B., G. T., A. G., R. G., A. A., A. K., and S. D. wrote the manuscript. The corresponding author had full access to all the data in the study and had final responsibility for the decision to submit for publication.

***Acknowledgments.*** The authors thank Bharat Yadav, Yagya Pokharel, Rateena Rajbhandari, Ronas Shakya, Mila Shakya, Dikshya Pant, Sudikchya Acharya, Nistha Shrestha, Ritu Shrestha, Manisha Risal, Rajana Pyatha, Anisha Prajapati, Anju Dheke, Anjana Tachamo, Sunil Khem, Neeta Gautam, Sarita Pyatha, Saheb Lal Chaudhary, Santosh Chaudhary, Bal Mukunda Neupane, Surendra Shrestha, Devina Twati, Rajendra Shrestha, Bijaya Karanjit, Muskuna Shrestha, Bidhya Laxmi Shrestha, Ramesh Duwa, Alisha Banjage, Jagadish Prasad Pandey, Manan Karki, Nancy Mittal, Abhusani Bhuju, Roshan Dhakal, Raj Kumar Thapa, Brijendra K Shah, Ramesh Bahadur Rokaya, Jagat Chaudhary, Ranjit Khatri, Bibek Raj Karki, Min Bahadur Rokaya, and Janak Bahadur Bohora; members of the data and safety monitoring committee (Christopher Parry, Nick White, Srijana Shrestha, Sue Lee); trial monitors (Le Thi Hoang Lan, Nguyen Thi Huyen Trang, Tran Thi Bich Ngoc); and the Department of Medicine, Department of Emergency and General Practice, and Department of Pediatrics, Patan Hospital, Patan Academy of Health Sciences, Lalitpur, Nepal. The authors also acknowledge the Department of Medicine, Family Medicine, Pediatrics, Microbiology and Pathology of Civil Services Hospital. Special thanks go to Dr Dirgharaj R. C., Dr Bimal Kumar Thapa, and Mr Madhav Ghimire for their support in Civil Service Hospital.

***Financial support.*** This work was supported by the Wellcome Trust (award number 106680/B/14/Z) and sponsored by the University of Oxford.

***Potential conflicts of interest.*** The authors: No reported conflicts of interest. All authors have submitted the ICMJE Form for Disclosure of Potential Conflicts of Interest.
