# Supplementary material for: Trimethoprim-sulfamethoxazole Versus Azithromycin for the Treatment of Undifferentiated Febrile Illness in Nepal: A Double-blind, Randomized, Placebo-controlled Trial
Source: Clin Infect Dis. 2020 Sep 29;73(7):e1478–86. doi: 10.1093/cid/ciaa1489 (PMC8492158; doi:10.1093/cid/ciaa1489)
Supplement: ciaa1489_suppl_Supplementary_Tables [file ciaa1489_suppl_supplementary_tables.docx]

Statistical analysis plan for the NUFIT 18NP Trial:

**Parallel group, double blind, 1:1 randomized controlled phase III trial of Co-trimoxazole versus Azithromycin in the treatment of Undifferentiated Febrile Illness in Nepal**

| Version Number & date: 18NP SAP 1.0 29MAR 2019 | | | |
| --- | --- | --- | --- |
| **Author** | **Position** | **Signature** | **Date** |
| Ronald Geskus | Trial Statistician | 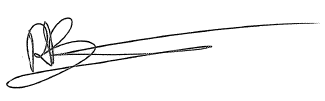 | 29^th^ March 2019 |
| Buddha Basnyat | OUCRU PI | 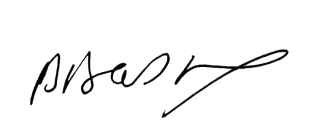 | 29^th^ March 2019 |
| Amit Arjyal | Investigator | 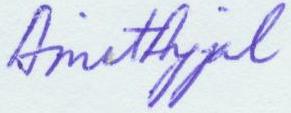 | 29^th^ March 2019 |
| Sunil Pokharel | Investigator | 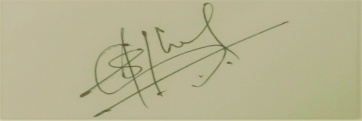 | 29^th^ March 2019 |

**Revision History**

| **Version** | **Author** | **Date** | **Reason for Revision** |
| --- | --- | --- | --- |
|  |  |  |  |
|  |  |  |  |
|  |  |  |  |

This document details the interim analysis for the randomised controlled clinical trial OUCRU 18NP (NCT02773407) conducted at Patan Hospital, Kathmandu as outlined in the trial protocol version 2.2 dated January 12^th^, 2018. It contains detailed definitions of the endpoints.

This analysis plan was written before un-blinding the trial data. Combination of blinding code and trial database are to be carried out only when the final analysis codes will be run on the statistical software. The final analysis was performed using R version 3.6.1 for Windows.

#### DATA SOURCES

The data-source for this analysis is the interim CliRes study database CTU18NP NUFIT which contains multiple data sets (eg. **DEMO** contains patients’ demographic information, **MH** contains medical history etc).

In this analysis plan, we refer to variables within datasets by separating them by a dot, e.g. **DEMO**.Age refers to the variable Age in dataset **DEMO** and contains the age of the patient.

All “blanks” must be considered as “the field is not applicable to the particular patient”.

#### TRIAL DESIGN AND SAMPLE SIZE

**Trial design**

Parallel group, double blinded, 1:1, randomized controlled phase III trial of co-trimoxazole versus azithromycin for the treatment of undifferentiated fever in Nepal.

**Sample size**

The target sample size for this trial is 330 subjects (165 per study group). This sample size is based on the sample size justification for 300 patients and an additional allowance of 10% to account for potential loss to follow-up and other deviations from the protocol assumptions as described in the protocol.

**Blinding**

Treatment allocation were concealed from the patient, investigators, study physicians, study nurses and other study staffs throughout the study and were hence was double blinded. Personnel involved in data entry and data checking were also blinded.

**Analysis**

The treatment allocations will remain blinded until the analysis plan and all data derivations have been finalized. Blinding codes and database will be handed to the statistician. Study statistician will run the code and carry out the analysis based on analysis plan.

#### 2. ANALYSIS POPULATIONS

There are three main populations defined:

1. **The intention to treat (ITT) population** consists of all patients who have been randomised to the trial and received at least one dose of study treatment (derived as **DAILYDRUG.**DrugAM=STUDY DRUG or if it is missing **DAILYDRUG.**DrugPM=STUDY DRUG at baseline **DAILYDRUG**.DAY=0). Analysis will be according to the randomized treatment arm (from randomization list). Patients who were mistakenly randomised or withdrew before the first dose of study treatment was given, will be excluded.

In case there is a mistake in randomization (for example, envelope opened incorrectly) or patient withdrawal from participation in the study or denial to take medication before the first dose of study drug will not be included in the analysis.

1. The **culture-confirmed population** consists of all patients with blood-culture confirmed enteric fever who received at least one dose of study treatment. Analysis will be according to the randomized treatment arm.

Culture-confirmed patients are identified in the database as those with *Salmonella Typhi* (**LAB**.Blood=*Salmonella* Typhi) or *Salmonella* Paratyphi A (**LAB**.Blood=*Salmonella* Paratyphi A) at baseline (**LAB.**Day = 0). Patient who received at least one study treatment is known by **DAILYDRUG.**DrugAM=STUDY DRUG or if it is missing by **DAILYDRUG.**DrugPM=STUDY DRUG at baseline **DAILYDRUG**.DAY=0.

1. The **culture negative population** consists of all patients with blood culture showing no growth or showing organisms other than *Salmonella* Typhi or *Salmonella* Paratyphi A on Day 0 who received at least one dose of study treatment. Analysis will be according to the randomized treatment arm.

Culture negative patients are identified in the database as those with no growth (**LAB**.Blood=NO GROWTH at baseline (**LAB.**Day = 0). Patient who received at least one study treatment is known **DAILYDRUG.**DrugAM=STUDY DRUG or if it is missing by **DAILYDRUG.**DrugPM=STUDY DRUG at baseline **DAILYDRUG**.DAY=0.

All analyses (unless mentioned otherwise) will be performed for the ITT, the per-protocol population, the culture-confirmed population, and in culture-negative patients.

#### 3. STUDY FLOW AND COMPLETENESS OF FOLLOW-UP

#### Study day 0 is defined as the date of the first dose of randomized treatment. We will summarize the following quantities related to follow-up duration by treatment arm:

#### Duration of daily follow-up – Median (IQR), number and proportion of patients with duration of daily follow-up visits for at least 7, 14, and 28 days based on DateAss column counted from DAY=0

#### Clinical data from daily follow-up visits are entered in dataset DAILYHIST, DAILYSE and DAILYEXAM.

#### Proportion of patients attending the day 7, 14, 28 and 63 follow-up visits (the window periods differ from the ones in the protocol)

#### Day 7 follow-up took place if data entered in DAILYEXAM.DateAss or DAILYHIST.DateAss or DAILYSE.DateAss between 7-1 and 7+3 after randomisation (earliest DateAss per individual)

#### Day 14 follow-up took place if data entered in DAILYEXAM.DateAss or DAILYHIST.DateAss or DAILYSE.DateAss between 14-3 and 14+7 after randomisation (earliest DateAss per individual) Day 28 follow-up took place if data entered in DAILYEXAM.DateAss or DAILYHIST.DateAss or DAILYSE.DateAss between 28-5 and 28+8 after randomisation (earliest DateAss per individual)

#### Day 63 follow-up took place if data entered in DAILYEXAM.DateAss or DAILYHIST.DateAss or DAILYSE.DateAss between 63-8 and 63+28 after randomisation (earliest DateAss per individual)

#### An unscheduled follow-up took place if data entered at DAILYEXAM.DateAss DAILYHIST.DateAss or DAILYSE.DateAss on any other day

#### The last day of follow up is from the latest DAILYEXAM.DateAss or DAILYHIST.DateAss, or DAILYSE.DateAss. In case of missing data, the data available from last follow-up will be used for the purpose of analysis.

1. **CALCULATION OF PRIMARY ENDPOINTS**

4.1 *Definition of Fever Clearance Time (FCT):* Fever clearance time (FCT) is the time from the first dose of a study drug until a first temperature ≤37·5^o^C (99.5^o^F) that continues for at least 48 hours.

*4.2. Derivation of FCT:*

A patients’ time and date of the first dose of treatment can be found in the row with **DAILYDRUG.**Day = 0. The date is given by **DAILYDRUG.**DateAss; the time is given by **DAILYDRUG.**TimeDrugAM or if it is missing by **DAILYDRUG.**TimeDrugPM.

#### (The first dose is given when the patient presents to hospital. If it is in the early afternoon, the patient will also get the second treatment later in the day. In such cases, the first dose is entered in AM column although given in PM time and the second dose is entered in PM column. The temperature values with missing time should be ignored for all temperature entries.)

All recorded temperature measurements from daily follow-up (recorded as **DAILYDRUG.**TempAM3, **DAILYDRUG.**TempAM4, **DAILYDRUG.**TempPM9, **DAILYDRUG.**TempPM10 and **DAILYDRUG.**TempPM11) will be included in the derivation of FCT. The time for temperature recordings for **DAILYDRUG.**TempAM3, **DAILYDRUG.**TempAM4, **DAILYDRUG.**TempPM9, **DAILYDRUG.**TempPM10 and **DAILYDRUG.**TempPM11 are available in **DAILYDRUG.**TempAM3a, **DAILYDRUG.**TempAM4a, **DAILYDRUG.**TempPM9a, **DAILYDRUG.**TempPM10a and **DAILYDRUG.**TempPM11a respectively. Temperature measurements taken after the documented temperature of ≤99.5^o^F for at least 48 hours will not be included in the analysis.

The time of fever clearance is based on the first time for which:

- Documented temperature ≤99.5^o^F at that time
- A documented temperature ≤99.5^o^F at least 48 hours later
- No documented temperatures >99.5°F between the two measurements above

The fever clearance time is interval censored between the latest time with fever and the first time of fever clearance.

Censoring for FCT calculation

Patients initiating rescue treatment (without prior fever clearance) in general will not be censored at that time point, i.e. we accept a hybrid fever clearance time based on multiple treatments. Only clinical treatment failures due to adverse events needing discontinuation of study drugs between DAY 0 and DAY 6 are censored at the time of rescue treatment initiation for the purpose of survival analysis for fever clearance. They are identified in the data set in case by case basis and reported to the trial statistician before un-blinding.

#### Because we need at least 48 hours of remaining in order to observe clearance, the censoring time is set at 48 hours before the observed censoring time in the calculations

1. **CALCULATION OF SECONDARY ENDPOINTS**

*Definitions of Secondary Endpoints:*

1) Treatment failure, defined as the occurrence of at least one of the following events:

a) Fever failure, defined by fever clearance time (FCT) >7 days (168 hours) post treatment initiation;

b) Requirement for rescue treatment as judged by the RMO and AP within Day 7 of treatment;

c) Blood culture positivity for *S.* Typhi or an *S.* Paratyphi on day 7 of treatment (microbiological failure);

d) Culture-confirmed or syndromic enteric fever relapse on/after day 8 and on/before day 28 of initiation of treatment;

e) The development of any complication (e.g. clinically significant bleeding, fall in the Glasgow Coma Score, perforation of the gastrointestinal tract and hospital admission) within 28 days after the initiation of treatment.

2) Time-to-treatment failure, defined as the time from the first dose of treatment until the date of the earliest failure event;

3) Adverse events (grade 3/4 adverse events, serious adverse events, adverse events of any grade leading to modification of study drug dose or interruption/early discontinuation).

**5.1.a Fever Failure - persistent fever at Day 7 of treatment**

All patients with a fever clearance time (FCT) > 7 days (168 hours) will be considered as having persistent fever. For these patients, the date of clinical treatment failure is day 7 (168 hours) of treatment.

*Derivation of FCT:*

A patients’ time and date of the first dose of treatment can be found in the row with **DAILYDRUG.**Day = 0. The date is given by **DAILYDRUG.**DateAss; the time is given by **DAILYDRUG.**TimeDrugAM or if it is missing by **DAILYDRUG.**TimeDrugPM.

All recorded temperature measurements from daily follow-up (recorded as **DAILYDRUG.**TempAM3, **DAILYDRUG.**TempAM4, **DAILYDRUG.**TempPM9, **DAILYDRUG.**TempPM10 and **DAILYDRUG.**TempPM11) will be included in the derivation of FCT. The time for temperature recordings for **DAILYDRUG.**TempAM3, **DAILYDRUG.**TempAM4, **DAILYDRUG.**TempPM9, **DAILYDRUG.**TempPM10 and **DAILYDRUG.**TempPM11 are available in **DAILYDRUG.**TempAM3a, **DAILYDRUG.**TempAM4a, **DAILYDRUG.**TempPM9a, **DAILYDRUG.**TempPM10a and **DAILYDRUG.**TempPM11a respectively. Temperature measurements taken after the documented temperature of ≤99.5^o^C for at least 48 hours will not be included in the analysis.

The fever failure is defined as the first time for which:

- Documented temperature >99.5^o^C at ≥168 hours from the first dose of study drug
- No documented temperature of ≤99.5^o^C for at least 48 hours during the first 168 hours

**5.1.b Clinical treatment failure - Need for ‘rescue’ treatment (with ceftriaxone, Ceftriaxone and Doxycycline, or any other antimicrobial)**

Patients who did not improve satisfactorily under their original treatment (clearance of fever and symptoms) were judged to have treatment failure by the study physician and their treatment was changed to ceftriaxone, ceftriaxone and doxycycline or any other antimicrobials.

Patients who develop adverse events needing discontinuation of study drug and administration of rescue treatment will also be judged as treatment failure.

Patients receiving rescue treatment before completion of study treatment are those with **DAILYDRUG.**DrugAM or **DAILYDRUG.**DrugPM = RESCUE TREATMENT **DAILYDRUG**.DAY=0,1,2,3,4,5 or 6. The corresponding date of rescue treatment initiation is **DAILYDRUG.**DateAss for the first entry DAILYDRUG.DrugAM=RESCUE TREATMENT or DAILYDRUG.DrugPM=RESCUE TREATMENT for each SUBJID. The corresponding time for rescue treatment initiation is **DAILYDRUG**.TimeDrugAM for the entry **DAILYDRUG**.DrugAM=RESCUE TREATMENT or if **DAILYDRUG**.DrugAM=STUDY DRUG, **DAILYDRUG**.Time DrugPM for the entry DAILYDRUG.DrugPM=RESCUE TREATMENT.

**5.1.c Microbiological failure**

Microbiological failure is defined as a blood culture positive for *S.* Typhi or *S.* Paratyphi A on Day=6 or Day=7 or Day=8 or Day9 or Day 10 of treatment among patients who are blood culture positive for *S.* Typhi or *S.* Paratyphi A on Day 0. Mismatching Salmonella serovars are also considered as microbiological failure. The date of microbiological failure is the date the blood culture has been obtained.

Blood culture results are recorded in dataset **LAB** and all cultures taken on day 6, day 7,day 8 ,day9 or day 10 **(LAB.**Day=6,7,8,9,10) will be included. Patients with microbiological failure are those who have a culture with S. Typhi (**LAB.**Blood=Salmonella Typhi) or S. Paratyphi A (**LAB.**Blood=Salmonella Paratyphi) at that day among patients whose initial blood culture is positive for S. Typhi or S. Paratyphi (**LAB.**Blood=Salmonella typhi or **LAB.**Blood=Salmonella paratyphi at **LAB.**DAY=0). Patients in culture-confirmed population with no blood culture on any of those days 6 to 10 will be excluded from the denominator for the calculation of microbiological failures.

**5.1.d Relapses**

Relapses are defined as the re-occurrence of symptoms and signs suggestive of enteric fever in patients who were initially categorised as successfully treated (not fever failure, not clinical treatment failure, not microbiological failure or no enteric fever related complications) and occurring on day 8 or later. This implies that relapses are only evaluated in patients who were initially categorised as successfully treated, i.e. patients with other treatment failures (i.e. fever failure, clinical treatment failure, microbiological failure, or enteric fever related complications) are not considered to have relapses. Also relapses are based on the clinical diagnosis without laboratory confirmation and it may be either culture positive or blood culture negative.

For the secondary endpoint, only relapses until day 28 of starting treatment will be regarded as treatment failures.

Relapses are either culture-confirmed enteric fever relapse or syndromic enteric fever relapses within 28 days of initiation of treatment. Mismatching *Salmonella* serovar are included as relapses.

Patients with syndromic relapses are those in the database with **DAILYSE**.SyndromicEF=1 at **DAILYSE**.DAY= 8 or later and **DAILYSE**.DAY=28 or earlier and **LAB**.Blood=NO GROWTH on that day. Blood culture confirmed relapses are those in the database with **DAILYSE**.SyndromicEF=1 at **DAILYSE**.DAY= 8 or later and **DAILYSE**.DAY=28 or earlier and **LAB**.Blood=”*Salmonella* Typhi” or “*Salmonella* Paratyphi A” on that day. The date of relapse is **DAILYSE**.DateAss for **DAILYSE**.SyndromicEF=1

**5.1.e Enteric fever related complications**

The development of any of the following enteric fever related complications within 28 days of initiation of treatment:

- clinically significant bleeding
- fall in the Glasgow Coma Score
- perforation of the gastrointestinal tract
- need for hospital admission

Complications are recorded as **DAILYSE**.Complications = 1 with corresponding **DALYSE**.DateAss applicable for **DAILYSE**.DAY=28 or earlier.

**5.2 Time-to-treatment failure**:

It is defined as the time from the first dose of treatment until the date of the earliest failure event.

All patients reaching one or more of the above components of treatment failure will be considered treatment failures. The date of treatment failure is the date of the earliest failure event of that patient. Patients without an event will be censored at the date of last follow-up in **DAILYHIST**.DAY or **DAILYSE**.DAY or **DAILYEXAM**.DAY.

**5.3 Adverse events**

Adverse events (AE) have been derived by study physicians who were blind to the treatment allocations. All adverse events are recorded in the **AE** dataset.

Tables will be generated to summarize both the overall numbers of adverse events for each SUBJID and the frequency of specific adverse events. The number of patients with at least one adverse event and the total number of adverse events will be summarized. Comparisons of the number of patients with each adverse event between the two arms will be done with Fisher’s exact test.

In addition, each adverse event of grade 3, 4 or 5 (**AE**.Grade=3, 4 or 5); adverse events requiring hospital admission (**AE**.HospAdmission=1); adverse events requiring study drug discontinuation (**AE**.Meddiscontinued=1) and adverse events persistent at the end of the study (**AE**.PersistentStudyEnd=1) will be summarized in the same way. All these will be summarized separately.

1. **ANALYSIS**
   1. **Analysis for Fever Clearance Time**

Fever clearance time (FCT) is defined in section 4.1. Interval-censored fever clearance time will be compared between the two groups based on a Weibull accelerated failure time model with the treatment arm as the only covariate. The distribution of the FCT over time in each treatment arm will be further visualized using the non-parametric maximum likelihood estimator (NPMLE).

Pre-defined subgroup analyses will be carried out in the ITT and the per-protocol population.

- Population (culture positive vs negative)
- Pathogen (*Salmonella* Typhi vs *Salmonella* Paratyphi A; in culture positives)

**6.2 Analyses of the treatment failure**

We will analyze both the time to treatment failure and the absolute risk of treatment failure until day 28. The former will be displayed with Kaplan-Meier curves and compared between the two groups with a Cox regression model with treatment as the only covariate. The Kaplan-Meier estimate on day 28 will be used as the estimate of the absolute risk of treatment failure and the comparison will be based on standard errors according to Greenwood’s formula.

In addition, the time to treatment failure and the absolute risk of treatment failure will be analyzed in the subgroups outlined below.

Pre-defined subgroups:

- Population (culture positive vs negative; in the ITT population)
- Pathogen (*Salmonella* Typhi vs *Salmonella* Paratyphi A; in culture positives)

**6.3 Analysis for relapses**

All recorded culture confirmed and syndromic relapses (as defined in Section 5.1.d) occurring in patients initially categorized as successfully treated will be summarized.

The time to relapse will be analyzed with survival analysis methods and relapses will be derived as defined in Section 5.1.d. Relapses occurring in patients meeting the definition for treatment failure prior to relapse (fever failure, clinical treatment failure, microbiological failure and enteric fever related complications) will not be counted and patients will be censored at the time of prior failure event All other patients will be censored at their last follow-up.

Planned analyses (in all patients and culture positive patients separately):

- Kaplan-Meier curves of the proportion of patients with relapse over time (until day 63)
- Comparison of the two groups (Cox regression with treatment as the only covariate) [relapse rate until day 28, and until day 63 separately]

1. **OTHER DESCRIPTIVE ANALYSES**

**Summary of baseline characteristics**

Baseline characteristic**s** will be summarized as median (IQR and range) for numeric data and n (%) for categorical data. No formal statistical comparison of baseline characteristics between the two study arms will be performed.

The following baseline characteristics will be summarized:

1. Patient details (**DEMO**): sex, age
2. Patient history (**MH**):

- Duration of illness (**MH.**DurationIllness)
- Symptoms-all variables (yes/no, not duration); **MH**.Fever, **MH**.Headache, **MH**.Anorexia, **MH**.Nausea, **MH**.Vomiting, **MH**.Diarrohea, **MH**.Constipation, **MH**.AbdoPain, **MH**.BlackStool, **MH**.Cough, **MH**.ChestPain, **MH**.ThroatDiscomfort, **MH**.Confusion, **MH**.Dizziness, **MH**.weakness, **MH**.Jointpain, **MH**.SkinRash, **MH**.OtherSymp, Each variable=0 means NO and Each variable≠0 means YES
- Past history-all variables; **MH**.PreviousTyphoid, **MH**.AcuteGastro, **MH**.TB, **MH**.ViralHepa, **MH**.FamilyTyphoid, **MH**.FamilyTB, **MH**.FamilyVIralHepa, **MH**.TyphoidVaccine, **MH**.Medication, **MH**.Allergy, **MH**.Treatment, **MH**.Antibiotics; Each variable=1 means YES and Each variable=2 means NO

1. **LAB** Investigations (hematology and biochemistry): **LAB**.HCT, **LAB**.WBC, **LAB**.Platelets, **LAB**.Stabs, **LAB**.Neutrophils, **LAB**.Lymphocytes, **LAB**.Monocytes, **LAB**.Eosinophils, **LAB**.Basophils, **LAB**.RBG, **LAB**.Creatinine, **LAB**.AST, **LAB**.ALT, **LAB**.UrineWBC, **LAB**.Blood, **LAB**.Stool

These will be summarized at baseline **LAB**.Day=0 along with follow-up blood measurements from **LAB**.

1. Household details from **HH**: All variables ; **HH**.NumberPeople, **HH**.NumberinKitchen, **HH**.SWClean, **HH**.OtherClean, **HH**.SWCook, **HH**.OtherCook, **HH**.SWDrink, **HH**.OtherDrink, **HH**.WaterTreat, **HH**.EatPlaceOften, **HH**.EatPlaceLast3W, **HH**.Other
2. Examinations from **PE**: all variables (except for free text fields)

**Randomization error**

In case there is a mistake in randomization (for example, envelope opened incorrectly) or patient withdrawal from participation in the study or denial to take medication before the first dose of study drug will not be included in the analysis.

Study number 080 and 081 have been removed as there was a randomization error. 080 is not included in the database. 081 is included in the database but will be removed from analysis. The randomization envelope 080 was overlooked and patient given drug from 081 for the first dose of treatment. The randomization envelope for 080 was also opened simultaneously by mistake and odd and even dose drugs form 080 and 081 got mixed. The mixing was determined after the first dose had been taken from 081. The patient was given alternative treatment. Therefore it was decided to cancel both randomization codes. The patient was explained about the randomization error. Therefore the patient was removed from the analysis. The randomization was resumed from 082 onwards.

**Treatment drop-outs**

Treatment drop outs are the patients who stop taking study medication during their treatment. They are identified in the dataset as at least three consecutive entries which are either BLANK or “TAB AZITHROMYCIN 500” or “LEVOFLOXACIN” in **DAILYDRUG**.DrugAM and**DAILYDRUG**.DrugPM considered together between DAY 0 and DAY6. Treatment drop-outs will be censored at the time of last “STUDY DRUG” entry in **DAILYDRUG**.DrugAM or **DAILYDRUG**.DrugPM reported in **DAILYDRUG.**TimeDrugAM or **DAILYDRUG.**TimeDrugPM.

**Proposed analyses**

ITT: All patients randomized ( except 080 and 081 who were mistakenly randomized)

Per protocol: All patients who are randomised and started on study intervention and complete study treatment or given rescue treatment before completion of study treatment. (To exclude treatment drop outs).
