# Supplementary material for: Trimethoprim-sulfamethoxazole Versus Azithromycin for the Treatment of Undifferentiated Febrile Illness in Nepal: A Double-blind, Randomized, Placebo-controlled Trial
Source: Clin Infect Dis. 2020 Sep 29;73(7):e1478–86. doi: 10.1093/cid/ciaa1489 (PMC8492158; doi:10.1093/cid/ciaa1489)
Supplement: ciaa1489_suppl_Supplementary_Material [file ciaa1489_suppl_supplementary_material.docx]

**Supplementary table 1:** Doses formulation for equivalent tablets in the two arms

| **Tablet 1** | **Tablet 2** | **Tablet 3** | **Tablet 4** |
| --- | --- | --- | --- |
| Azithromycin 800 mg | Azithromycin 400 mg | Azithromycin 200 mg | Azithromycin 100 mg |
| Co-trimoxazole 1200 mg | Co-trimoxazole 600 mg | Co-trimoxazole 300 mg | Co-trimoxazole 150 mg |
| Placebo tablet 1 (equivalent to Azithromycin 800 mg) | Placebo tablet 2 (equivalent to Azithromycin 400 mg) | Placebo tablet 3 (equivalent to Azithromycin 200 mg) | Placebo tablet 4 (equivalent to Azithromycin 100 mg) |
|  |  |  |  |

**Supplementary table 2:** Placebo composition and its content

| **Name of Ingredients** | **Weight in different tablets** | | | |
| --- | --- | --- | --- | --- |
|  | **100mg** | **200mg** | **400mg** | **800mg** |
| Mannitol | 104.8 | 109.6 | 219.2 | 238.4 |
| Dibasic calcium phosphate | 50 | 55 | 125 | 245 |
| Starch | 30.1 | 35 | 185 | 200 |
| PVP K-30 | 6.9 | 8.5 | 21.5 | 34 |
| Isopropyl alcohol | 0 | 0 | 0 | 0 |
| MCC Ph 102 | 83.15 | 117.5 | 212.2 | 274 |
| Sodium Lauryl phosphate | 10.85 | 15.5 | 30.1 | 47.6 |
| Cross carmellose so | 15.5 | 18.5 | 43 | 68 |
| P. Talk | 6.2 | 7.4 | 17.2 | 27.2 |
| Magnesium Stearate | 2.5 | 3 | 6.8 | 10.8 |
| MCC Ph 101 |  |  |  | 215 |
| Total | 310 | 370 | 860 | 1360 |

.

|  | **Azithromycin arm**  **Supplementary table 3:** Drug dose for azithromycin and co-trimoxazole according to body weight | | | | | | | | | | | |
| --- | --- | --- | --- | --- | --- | --- | --- | --- | --- | --- | --- | --- |
|  |  |  | **Odd number dose in Azithro arm** | | | | **Even number dose in Azithro arm** | | | |  |  |
| **Weight in kg** | **Dose Azithromycin (mg/kg/day)** | **Daily Total Azithromycin** | **800 mg tablet** | **400 mg tablet** | **200 mg tablet** | **100 mg tablet** | **Placebo 1 tab** | **Placebo 2 tab** | **Placebo 3 tab** | **Placebo 4 tab** | **actual dose of azithro/day** | **actual dose of azithro/kg/day** |
| 10 | 20 | 200 | 0 | 0 | 1 | 0 | 0 | 0 | 1 | 0 | 200 | 20 |
| 11 | 20 | 220 | 0 | 0 | 1 | 0 | 0 | 0 | 1 | 0 | 200 | 18.18181818 |
| 12 | 20 | 240 | 0 | 0 | 1 | 0 | 0 | 0 | 1 | 0 | 200 | 16.66666667 |
| 13 | 20 | 260 | 0 | 0 | 1 | 1 | 0 | 0 | 1 | 1 | 300 | 23.07692308 |
| 14 | 20 | 280 | 0 | 0 | 1 | 1 | 0 | 0 | 1 | 1 | 300 | 21.42857143 |
| 15 | 20 | 300 | 0 | 0 | 1 | 1 | 0 | 0 | 1 | 1 | 300 | 20 |
| 16 | 20 | 320 | 0 | 0 | 1 | 1 | 0 | 0 | 1 | 1 | 300 | 18.75 |
| 17 | 20 | 340 | 0 | 0 | 1 | 1 | 0 | 0 | 1 | 1 | 300 | 17.64705882 |
| 18 | 20 | 360 | 0 | 1 | 0 | 0 | 0 | 1 | 0 | 0 | 400 | 22.22222222 |
| 19 | 20 | 380 | 0 | 1 | 0 | 0 | 0 | 1 | 0 | 0 | 400 | 21.05263158 |
| 20 | 20 | 400 | 0 | 1 | 0 | 0 | 0 | 1 | 0 | 0 | 400 | 20 |
| 21 | 20 | 420 | 0 | 1 | 0 | 0 | 0 | 1 | 0 | 0 | 400 | 19.04761905 |
| 22 | 20 | 440 | 0 | 1 | 0 | 0 | 0 | 1 | 0 | 0 | 400 | 18.18181818 |
| 23 | 20 | 460 | 0 | 1 | 0 | 1 | 0 | 1 | 0 | 1 | 500 | 21.73913043 |
| 24 | 20 | 480 | 0 | 1 | 0 | 1 | 0 | 1 | 0 | 1 | 500 | 20.83333333 |
| 25 | 20 | 500 | 0 | 1 | 0 | 1 | 0 | 1 | 0 | 1 | 500 | 20 |
| 26 | 20 | 520 | 0 | 1 | 0 | 1 | 0 | 1 | 0 | 1 | 500 | 19.23076923 |
| 27 | 20 | 540 | 0 | 1 | 0 | 1 | 0 | 1 | 0 | 1 | 500 | 18.51851852 |
| 28 | 20 | 560 | 0 | 1 | 1 | 0 | 0 | 1 | 1 | 0 | 600 | 21.42857143 |
| 29 | 20 | 580 | 0 | 1 | 1 | 0 | 0 | 1 | 1 | 0 | 600 | 20.68965517 |
| 30 | 20 | 600 | 0 | 1 | 1 | 0 | 0 | 1 | 1 | 0 | 600 | 20 |
| 31 | 20 | 620 | 0 | 1 | 1 | 0 | 0 | 1 | 1 | 0 | 600 | 19.35483871 |
| 32 | 20 | 640 | 0 | 1 | 1 | 0 | 0 | 1 | 1 | 0 | 600 | 18.75 |
| 33 | 20 | 660 | 0 | 1 | 1 | 1 | 0 | 1 | 1 | 1 | 700 | 21.21212121 |
| 34 | 20 | 680 | 0 | 1 | 1 | 1 | 0 | 1 | 1 | 1 | 700 | 20.58823529 |
| 35 | 20 | 700 | 0 | 1 | 1 | 1 | 0 | 1 | 1 | 1 | 700 | 20 |
| 36 | 20 | 720 | 0 | 1 | 1 | 1 | 0 | 1 | 1 | 1 | 700 | 19.44444444 |
| 37 | 20 | 740 | 0 | 1 | 1 | 1 | 0 | 1 | 1 | 1 | 700 | 18.91891892 |
| 38 | 20 | 760 | 1 | 0 | 0 | 0 | 1 | 0 | 0 | 0 | 800 | 21.05263158 |
| 39 | 20 | 780 | 1 | 0 | 0 | 0 | 1 | 0 | 0 | 0 | 800 | 20.51282051 |
| 40 | 20 | 800 | 1 | 0 | 0 | 0 | 1 | 0 | 0 | 0 | 800 | 20 |
| 41 | 20 | 820 | 1 | 0 | 0 | 0 | 1 | 0 | 0 | 0 | 800 | 19.51219512 |
| 42 | 20 | 840 | 1 | 0 | 0 | 0 | 1 | 0 | 0 | 0 | 800 | 19.04761905 |
| 43 | 20 | 860 | 1 | 0 | 0 | 1 | 1 | 0 | 0 | 1 | 900 | 20.93023256 |
| 44 | 20 | 880 | 1 | 0 | 0 | 1 | 1 | 0 | 0 | 1 | 900 | 20.45454545 |
| 45 | 20 | 900 | 1 | 0 | 0 | 1 | 1 | 0 | 0 | 1 | 900 | 20 |
| 46 | 20 | 920 | 1 | 0 | 0 | 1 | 1 | 0 | 0 | 1 | 900 | 19.56521739 |
| 47 | 20 | 940 | 1 | 0 | 0 | 1 | 1 | 0 | 0 | 1 | 900 | 19.14893617 |
| 48 | 20 | 960 | 1 | 0 | 1 | 0 | 1 | 0 | 1 | 0 | 1000 | 20.83333333 |
| 49 | 20 | 980 | 1 | 0 | 1 | 0 | 1 | 0 | 1 | 0 | 1000 | 20.40816327 |
| 50 | 20 | 1000 | 1 | 0 | 1 | 0 | 1 | 0 | 1 | 0 | 1000 | 20 |
| 51 | 20 | 1020 | 1 | 0 | 1 | 0 | 1 | 0 | 1 | 0 | 1000 | 19.60784314 |
| 52 | 20 | 1040 | 1 | 0 | 1 | 0 | 1 | 0 | 1 | 0 | 1000 | 19.23076923 |
| 53 | 20 | 1060 | 1 | 0 | 1 | 0 | 1 | 0 | 1 | 0 | 1000 | 18.86792453 |
| 54 | 20 | 1080 | 1 | 0 | 1 | 0 | 1 | 0 | 1 | 0 | 1000 | 18.51851852 |
| 55 | 20 | 1100 | 1 | 0 | 1 | 0 | 1 | 0 | 1 | 0 | 1000 | 18. 18181818 |
| 56 | 20 | 1120 | 1 | 0 | 1 | 0 | 1 | 0 | 1 | 0 | 1000 | 17.85714286 |
| 57 | 20 | 1140 | 1 | 0 | 1 | 0 | 1 | 0 | 1 | 0 | 1000 | 17.54385965 |
| 58 | 20 | 1160 | 1 | 0 | 1 | 0 | 1 | 0 | 1 | 0 | 1000 | 17. 24137931 |
| 59 | 20 | 1180 | 1 | 0 | 1 | 0 | 1 | 0 | 1 | 0 | 1000 | 16.94915254 |
| 60 | 20 | 1200 | 1 | 0 | 1 | 0 | 1 | 0 | 1 | 0 | 1000 | 16.66666667 |
| 61 | 20 | 1220 | 1 | 0 | 1 | 0 | 1 | 0 | 1 | 0 | 1000 | 16.39344262 |
| 62 | 20 | 1240 | 1 | 0 | 1 | 0 | 1 | 0 | 1 | 0 | 1000 | 16.12903226 |
| 63 | 20 | 1260 | 1 | 0 | 1 | 0 | 1 | 0 | 1 | 0 | 1000 | 15.87301587 |
| 64 | 20 | 1280 | 1 | 0 | 1 | 0 | 1 | 0 | 1 | 0 | 1000 | 15. 625 |
| 65 | 20 | 1300 | 1 | 0 | 1 | 0 | 1 | 0 | 1 | 0 | 1000 | 15.38461538 |
| 66 | 20 | 1320 | 1 | 0 | 1 | 0 | 1 | 0 | 1 | 0 | 1000 | 15.15151515 |
| 67 | 20 | 1340 | 1 | 0 | 1 | 0 | 1 | 0 | 1 | 0 | 1000 | 14.92537313 |
| 68 | 20 | 1360 | 1 | 0 | 1 | 0 | 1 | 0 | 1 | 0 | 1000 | 14.70588235 |
| 69 | 20 | 1380 | 1 | 0 | 1 | 0 | 1 | 0 | 1 | 0 | 1000 | 14.49275362 |
| 70 | 20 | 1400 | 1 | 0 | 1 | 0 | 1 | 0 | 1 | 0 | 1000 | 14.28571429 |
| 71 | 20 | 1420 | 1 | 0 | 1 | 0 | 1 | 0 | 1 | 0 | 1000 | 14.08450704 |
| 72 | 20 | 1440 | 1 | 0 | 1 | 0 | 1 | 0 | 1 | 0 | 1000 | 13.88888889 |
| 73 | 20 | 1460 | 1 | 0 | 1 | 0 | 1 | 0 | 1 | 0 | 1000 | 13.69863014 |
| 74 | 20 | 1480 | 1 | 0 | 1 | 0 | 1 | 0 | 1 | 0 | 1000 | 13.51351351 |
| 75 | 20 | 1500 | 1 | 0 | 1 | 0 | 1 | 0 | 1 | 0 | 1000 | 13.33333333 |
| 76 | 20 | 1520 | 1 | 0 | 1 | 0 | 1 | 0 | 1 | 0 | 1000 | 13.15789474 |
| 77 | 20 | 1540 | 1 | 0 | 1 | 0 | 1 | 0 | 1 | 0 | 1000 | 12.98701299 |
| 78 | 20 | 1560 | 1 | 0 | 1 | 0 | 1 | 0 | 1 | 0 | 1000 | 12.82051282 |
| 79 | 20 | 1580 | 1 | 0 | 1 | 0 | 1 | 0 | 1 | 0 | 1000 | 12.65822785 |
| 80 | 20 | 1600 | 1 | 0 | 1 | 0 | 1 | 0 | 1 | 0 | 1000 | 12.5 |
| 81 | 20 | 1620 | 1 | 0 | 1 | 0 | 1 | 0 | 1 | 0 | 1000 | 12.34567901 |
| 82 | 20 | 1640 | 1 | 0 | 1 | 0 | 1 | 0 | 1 | 0 | 1000 | 12.19512195 |
| 83 | 20 | 1660 | 1 | 0 | 1 | 0 | 1 | 0 | 1 | 0 | 1000 | 12.04819277 |
| 84 | 20 | 1680 | 1 | 0 | 1 | 0 | 1 | 0 | 1 | 0 | 1000 | 11.9047619 |
| 85 | 20 | 1700 | 1 | 0 | 1 | 0 | 1 | 0 | 1 | 0 | 1000 | 11.76470588 |
| 86 | 20 | 1720 | 1 | 0 | 1 | 0 | 1 | 0 | 1 | 0 | 1000 | 11.62790698 |
| 87 | 20 | 1740 | 1 | 0 | 1 | 0 | 1 | 0 | 1 | 0 | 1000 | 11.49425287 |
| 88 | 20 | 1760 | 1 | 0 | 1 | 0 | 1 | 0 | 1 | 0 | 1000 | 11.36363636 |
| 89 | 20 | 1780 | 1 | 0 | 1 | 0 | 1 | 0 | 1 | 0 | 1000 | 11.23595506 |
| 90 | 20 | 1800 | 1 | 0 | 1 | 0 | 1 | 0 | 1 | 0 | 1000 | 11.11111111 |
| 91 | 20 | 1820 | 1 | 0 | 1 | 0 | 1 | 0 | 1 | 0 | 1000 | 10.98901099 |
| 92 | 20 | 1840 | 1 | 0 | 1 | 0 | 1 | 0 | 1 | 0 | 1000 | 10.86956522 |
| 93 | 20 | 1860 | 1 | 0 | 1 | 0 | 1 | 0 | 1 | 0 | 1000 | 10.75268817 |
| 94 | 20 | 1880 | 1 | 0 | 1 | 0 | 1 | 0 | 1 | 0 | 1000 | 10.63829787 |
| 95 | 20 | 1900 | 1 | 0 | 1 | 0 | 1 | 0 | 1 | 0 | 1000 | 10.52631579 |
| 96 | 20 | 1920 | 1 | 0 | 1 | 0 | 1 | 0 | 1 | 0 | 1000 | 10.41666667 |
| 97 | 20 | 1940 | 1 | 0 | 1 | 0 | 1 | 0 | 1 | 0 | 1000 | 10.30927835 |
| 98 | 20 | 1960 | 1 | 0 | 1 | 0 | 1 | 0 | 1 | 0 | 1000 | 10.20408163 |
| 99 | 20 | 1980 | 1 | 0 | 1 | 0 | 1 | 0 | 1 | 0 | 1000 | 10.1010101 |
| 100 | 20 | 2000 | 1 | 0 | 1 | 0 | 1 | 0 | 1 | 0 | 1000 | 10 |

| **Co-trimoxazole arm** | | | | | | | | |
| --- | --- | --- | --- | --- | --- | --- | --- | --- |
|  |  |  | **Odd/even number dose in Cotrim arm** | | | |  |  |
| **Dose Cotrim (T:S=1:5) (mg/kg/day)** | **Daily Total Cotrim** | **Cotrim per dose** | **1200 mg tab** | **600 mg tab** | **300 mg tab** | **150 mg tab** | **actual dose of cotrim/day** | **actual dose of cotrim/kg/day** |
| 60 | 600 | 300 | 0 | 0 | 1 | 0 | 600 | 60 |
| 60 | 660 | 330 | 0 | 0 | 1 | 0 | 600 | 54.54545455 |
| 60 | 720 | 360 | 0 | 0 | 1 | 0 | 600 | 50 |
| 60 | 780 | 390 | 0 | 0 | 1 | 1 | 900 | 69.23076923 |
| 60 | 840 | 420 | 0 | 0 | 1 | 1 | 900 | 64.28571429 |
| 60 | 900 | 450 | 0 | 0 | 1 | 1 | 900 | 60 |
| 60 | 960 | 480 | 0 | 0 | 1 | 1 | 900 | 56.25 |
| 60 | 1020 | 510 | 0 | 0 | 1 | 1 | 900 | 52.94117647 |
| 60 | 1080 | 540 | 0 | 1 | 0 | 0 | 1200 | 66.6666667 |
| 60 | 1140 | 570 | 0 | 1 | 0 | 0 | 1200 | 63.15789474 |
| 60 | 1200 | 600 | 0 | 1 | 0 | 0 | 1200 | 60 |
| 60 | 1260 | 630 | 0 | 1 | 0 | 0 | 1200 | 57.14285714 |
| 60 | 1320 | 660 | 0 | 1 | 0 | 0 | 1200 | 54.54545455 |
| 60 | 1380 | 690 | 0 | 1 | 0 | 1 | 1500 | 65.2173913 |
| 60 | 1440 | 720 | 0 | 1 | 0 | 1 | 1500 | 62.5 |
| 60 | 1500 | 750 | 0 | 1 | 0 | 1 | 1500 | 60 |
| 60 | 1560 | 780 | 0 | 1 | 0 | 1 | 1500 | 57.69230769 |
| 60 | 1620 | 810 | 0 | 1 | 0 | 1 | 1500 | 55.55555556 |
| 60 | 1680 | 840 | 0 | 1 | 1 | 0 | 1800 | 64.28571429 |
| 60 | 1740 | 870 | 0 | 1 | 1 | 0 | 1800 | 62.06896552 |
| 60 | 1800 | 900 | 0 | 1 | 1 | 0 | 1800 | 60 |
| 60 | 1860 | 930 | 0 | 1 | 1 | 0 | 1800 | 58.06451613 |
| 60 | 1920 | 960 | 0 | 1 | 1 | 0 | 1800 | 56.25 |
| 60 | 1980 | 990 | 0 | 1 | 1 | 1 | 2100 | 63.63636364 |
| 60 | 2040 | 1020 | 0 | 1 | 1 | 1 | 2100 | 61.76470588 |
| 60 | 2100 | 1050 | 0 | 1 | 1 | 1 | 2100 | 60 |
| 60 | 2160 | 1080 | 0 | 1 | 1 | 1 | 2100 | 58.33333333 |
| 60 | 2220 | 1110 | 0 | 1 | 1 | 1 | 2100 | 56.75675676 |
| 60 | 2280 | 1140 | 1 | 0 | 0 | 0 | 2400 | 63.15789474 |
| 60 | 2340 | 1170 | 1 | 0 | 0 | 0 | 2400 | 61.53846154 |
| 60 | 2400 | 1200 | 1 | 0 | 0 | 0 | 2400 | 60 |
| 60 | 2460 | 1230 | 1 | 0 | 0 | 0 | 2400 | 58.53658537 |
| 60 | 2520 | 1260 | 1 | 0 | 0 | 0 | 2400 | 57.14285714 |
| 60 | 2580 | 1290 | 1 | 0 | 0 | 1 | 2700 | 62.79069767 |
| 60 | 2640 | 1320 | 1 | 0 | 0 | 1 | 2700 | 61.36363636 |
| 60 | 2700 | 1350 | 1 | 0 | 0 | 1 | 2700 | 60 |
| 60 | 2760 | 1380 | 1 | 0 | 0 | 1 | 2700 | 58.69565217 |
| 60 | 2820 | 1410 | 1 | 0 | 0 | 1 | 2700 | 57.44680851 |
| 60 | 2880 | 1440 | 1 | 0 | 1 | 0 | 3000 | 62.5 |
| 60 | 2940 | 1470 | 1 | 0 | 1 | 0 | 3000 | 61.2244898 |
| 60 | 3000 | 1500 | 1 | 0 | 1 | 0 | 3000 | 60 |
| 60 | 3060 | 1530 | 1 | 0 | 1 | 0 | 3000 | 58.82352941 |
| 60 | 3120 | 1560 | 1 | 0 | 1 | 0 | 3000 | 57.69230769 |
| 60 | 3180 | 1590 | 1 | 0 | 1 | 0 | 3000 | 56.60377358 |
| 60 | 3240 | 1620 | 1 | 0 | 1 | 0 | 3000 | 55.55555556 |
| 60 | 3300 | 1650 | 1 | 0 | 1 | 0 | 3000 | 54.54545455 |
| 60 | 3360 | 1680 | 1 | 0 | 1 | 0 | 3000 | 53.57142857 |
| 60 | 3420 | 1710 | 1 | 0 | 1 | 0 | 3000 | 52.63157895 |
| 60 | 3480 | 1740 | 1 | 0 | 1 | 0 | 3000 | 51.72413793 |
| 60 | 3540 | 1770 | 1 | 0 | 1 | 0 | 3000 | 50.84745763 |
| 60 | 3600 | 1800 | 1 | 0 | 1 | 0 | 3000 | 50 |
| 60 | 3660 | 1830 | 1 | 0 | 1 | 0 | 3000 | 49.18032787 |
| 60 | 3720 | 1860 | 1 | 0 | 1 | 0 | 3000 | 48.38709677 |
| 60 | 3780 | 1890 | 1 | 0 | 1 | 0 | 3000 | 47.61904762 |
| 60 | 3840 | 1920 | 1 | 0 | 1 | 0 | 3000 | 46.875 |
| 60 | 3900 | 1950 | 1 | 0 | 1 | 0 | 3000 | 46.15384615 |
| 60 | 3960 | 1980 | 1 | 0 | 1 | 0 | 3000 | 45.45454545 |
| 60 | 4020 | 2010 | 1 | 0 | 1 | 0 | 3000 | 44.7761194 |
| 60 | 4080 | 2040 | 1 | 0 | 1 | 0 | 3000 | 44.11764706 |
| 60 | 4140 | 2070 | 1 | 0 | 1 | 0 | 3000 | 43.47826087 |
| 60 | 4200 | 2100 | 1 | 0 | 1 | 0 | 3000 | 42.85714286 |
| 60 | 4260 | 2130 | 1 | 0 | 1 | 0 | 3000 | 42.25352113 |
| 60 | 4320 | 2160 | 1 | 0 | 1 | 0 | 3000 | 41.66666667 |
| 60 | 4380 | 2190 | 1 | 0 | 1 | 0 | 3000 | 41.09589041 |
| 60 | 4440 | 2220 | 1 | 0 | 1 | 0 | 3000 | 40.54054054 |
| 60 | 4500 | 2250 | 1 | 0 | 1 | 0 | 3000 | 40 |
| 60 | 4560 | 2280 | 1 | 0 | 1 | 0 | 3000 | 39.47368421 |
| 60 | 4620 | 2310 | 1 | 0 | 1 | 0 | 3000 | 38.96103896 |
| 60 | 4680 | 2340 | 1 | 0 | 1 | 0 | 3000 | 38.46153846 |
| 60 | 4740 | 2370 | 1 | 0 | 1 | 0 | 3000 | 37.97468354 |
| 60 | 4800 | 2400 | 1 | 0 | 1 | 0 | 3000 | 37.5 |
| 60 | 4860 | 2430 | 1 | 0 | 1 | 0 | 3000 | 37.03703704 |
| 60 | 4920 | 2460 | 1 | 0 | 1 | 0 | 3000 | 36.58536585 |
| 60 | 4980 | 2490 | 1 | 0 | 1 | 0 | 3000 | 36.14457831 |
| 60 | 5040 | 2520 | 1 | 0 | 1 | 0 | 3000 | 35.71428571 |
| 60 | 5100 | 2550 | 1 | 0 | 1 | 0 | 3000 | 35.29411765 |
| 60 | 5160 | 2580 | 1 | 0 | 1 | 0 | 3000 | 34.88372093 |
| 60 | 5220 | 2610 | 1 | 0 | 1 | 0 | 3000 | 34.48275862 |
| 60 | 5280 | 2640 | 1 | 0 | 1 | 0 | 3000 | 34.09090909 |
| 60 | 5340 | 2670 | 1 | 0 | 1 | 0 | 3000 | 33.70786517 |
| 60 | 5400 | 2700 | 1 | 0 | 1 | 0 | 3000 | 33.33333333 |
| 60 | 5460 | 2730 | 1 | 0 | 1 | 0 | 3000 | 32.96703297 |
| 60 | 5520 | 2760 | 1 | 0 | 1 | 0 | 3000 | 32.60869565 |
| 60 | 5580 | 2790 | 1 | 0 | 1 | 0 | 3000 | 32.25806452 |
| 60 | 5640 | 2820 | 1 | 0 | 1 | 0 | 3000 | 31.91489362 |
| 60 | 5700 | 2850 | 1 | 0 | 1 | 0 | 3000 | 31.57894737 |
| 60 | 5760 | 2880 | 1 | 0 | 1 | 0 | 3000 | 31.25 |
| 60 | 5820 | 2910 | 1 | 0 | 1 | 0 | 3000 | 30.92783505 |
| 60 | 5880 | 2940 | 1 | 0 | 1 | 0 | 3000 | 30.6122449 |
| 60 | 5940 | 2970 | 1 | 0 | 1 | 0 | 3000 | 30.3030303 |
| 60 | 6000 | 3000 | 1 | 0 | 1 | 0 | 3000 | 30 |

|  | **STUDY PERIOD** | | | | | | | | | | | | | |
| --- | --- | --- | --- | --- | --- | --- | --- | --- | --- | --- | --- | --- | --- | --- |
| **Study Day** | **Enrolment (DO)** | **D**  **1** | **D**  **2** | **D**  **3** | **D**  **4** | **D**  **5** | **D**  **6** | **D**  **7** | **D**  **8** | **D**  **9** | **D**  **10** | **D**  **14** | **D**  **28** | **D**  **63** |
| **ENROLMENT:** |  | | | | | | | | | | | | | |
| Eligibility screen | X |  |  |  |  |  |  |  |  |  |  |  |  |  |
| Informed consent | X |  |  |  |  |  |  |  |  |  |  |  |  |  |
| Randomization | X |  |  |  |  |  |  |  |  |  |  |  |  |  |
| Allocation | X |  |  |  |  |  |  |  |  |  |  |  |  |  |
| **INTERVENTIONS:** |  | | | | | | | | | | | | | |
| Study Drug Administration | X  X | X  X | X  X | X  X | X  X | X  X | X  X | X |  |  |  |  |  |  |
| **ASSESMENTS:** |  | | | | | | | | | | | | | |
| Hospital Visits and Physical Exam | X |  |  |  |  |  |  | X |  |  |  |  | X | X |
| Patient follow up by CMA by home visits |  |  | X |  | X |  |  |  |  |  |  |  |  |  |
| Record of Symptoms, any AE/SE and complications | X | Ჲ | Ჲ | Ჲ | Ჲ | Ჲ | Ჲ | X | Ჲ | Ჲ | Ჲ | X |  |  |
| Temperature | X  Ჲ | Ჲ  Ჲ | Ჲ  Ჲ | Ჲ  Ჲ | Ჲ  Ჲ | Ჲ  Ჲ | Ჲ  Ჲ | Ჲ  Ჲ | Ჲ  Ჲ | Ჲ  Ჲ | Ჲ  Ჲ | X |  |  |
| Haematology(1ml)  (Hct, WBC/diff, plt) | X |  |  |  |  |  |  | X |  |  |  |  |  |  |
| Biochemistry(1ml)  (RBG/SGOT/SGPT/Creatinine) | X |  |  |  |  |  |  | X |  |  |  |  |  |  |
| Urine for routine examination | X |  |  |  |  |  |  |  |  |  |  |  |  |  |
| Blood for Culture (3ml/5-8ml) | X |  |  |  |  |  |  | (X) |  |  |  |  |  |  |
| Blood and plasma sample for storage | X |  |  |  |  |  |  | X |  |  |  | X | X | X |
| Urine sample for storage  **Supplementary table 4 :** follow-up intervals and the assessment schedule used in the study. | X |  |  |  |  |  |  |  |  |  |  |  |  |  |
| Stool for culture | X |  |  |  |  |  |  | (X) |  |  |  | (X) | (X) | (X) |
| Further diagnostics for patients with culture negative (blood sample at enrollment) UFI | X* |  |  |  |  |  |  |  |  |  |  |  | X* |  |

**Supplementary Table 5:** Power for the overall population, culture-negative patients and culture-positive patients.

| **Total sample size in both groups (culture-positive/-negative)** | **All patients (ITT): power for superiority of azithromycin** | **Culture-negative patients: power for superiority of azithromycin** | **Culture-positive patients: power for “non-inferiority” of azithromycin^a^** |
| --- | --- | --- | --- |
| 300 (150/150) | 80% | 89% | 96% |
| 300 (100/200) | 92% | 95% | 86% |

Probability that the 95% confidence interval for the effect of azithromycin excludes the possibility that azithromycin is associated with a 1.5-fold slower fever clearance time (FCT) in culture-positive patients.

*ITT* intention-to-treat

**Supplementary Table 6:** Heterogeneity test for interval censored fever clearance time for other subgroups

| Stratum | Co-trimoxazole  N: Median | Azithromycin  N: Median | Acceleration Factor (95%CI) p-value | Test for effect heterogeneity  p-value interaction test |
| --- | --- | --- | --- | --- |
| All patients | 163:2.42(0.83,5.65) | 163:2.11(0.84,4.36) | 1.29(0.99,1.68);p=0.06 |  |
| **Culture** |  |  |  | 0.09 |
| Negatives | 112:1.97(0.59,5.11) | 120:1.53(0.55,3.42) | 1.49(1.05,2.10);p=0.024 | |
| Positives | 45:3.76 (1.91,6.42) | 42:4.74(3.06,6.71) | 0.96(0.72,1.29);p=0.81 |  |
| **Pathogen** |  |  |  | 0.82 |
| *S*. Paratyphi | 4:3.71(2.74,4.71) | 3:4.52(3.96,5.01) | 0.94(0.63,1.41);p=0.77 |  |
| *S*.Typhi | 41:3.79(1.88,6.58) | 39:4.76(3.03,6.81) | 0.97(0.71,1.33);p=0.86 |  |
| **Age Category** |  |  |  | 0.98 |
| <14 | 22:2.69(1.05,5.62) | 28:2.34(1.06,4.35) | 1.30(0.75,2.27);p=0.35 |  |
| ≥14 | 140:2.35(0.78,5.61) | 134:2.04(0.78,4.34) | 1.28(0.95,1.73);p=0.10 |  |
| **Age (culture positive)** | |  |  | 0.74 |
| <14 | 7:3.89(2.47,5.58) | 8:3.39(1.96,5.2) | 1.06(0.58,1.96);p=0.85 |  |
| ≥14 | 38:3.76(1.86,6.56) | 34:5.14(3.44,7.04) | 0.93(0.67,1.30);p=0.69 |  |
| **Sex** |  |  |  | 0.68 |
| Male | 113:2.38(0.83,5.43) | 104:1.78(0.64,3.98) | 1.36(0.98,1.91);p=0.07 |  |
| Female | 50:2.5(0.8,6.43) | 59:2.75(1.31,4.92) | 1.22(0.81,1.85);p=0.34 |  |
| **Sex(culture positive)** |  |  |  | 0.2 |
| Male | 28:3.53(1.8,6) | 22:5.53(3.9,7.28) | 082(0.57,1.19);p=0.30 |  |
| Female | 17:4.11(2.11,6.96) | 20:3.97(2.36,5.98) | 1.18(0.75,1.85);p=0.48 |  |
| **MIC azithromycin** |  |  |  | 0.65 |
| ≤3 | 37:3.76(1.91,6.4) | 32:4.29(2.72,6.13) | 1.05(0.75,1.47);p=0.79 |  |
| >3 | 7:4.85(2.87,7.34) | 7:6.13(4.17,8.32) | 0.90(0.51,1.60);p=0.72 |  |
| **MIC cotrimoxazole** |  |  |  | 0.43 |
| ≤0.047 | 28:4.47(2.32,7.51) | 27:4.47(2.63,6.79) | 1.10(0.75,1.63);p=0.62 |  |
| >0.047 | 16:3.19(1.73,5.17) | 11:4.76(3.6,5.93) | 0.88(0.56,1.41);p=0.60 |  |

**Supplementary Table 7:** Minimum inhibitory concentration of organism in the culture-confirmed population

| **Characteristics** | **n** | **Summary statistics of all patients (N=87)** | **n** | **Summary statistics of *S.* PARATYPHI "A" (N=7)** | **n** | **Summary statistics of *S.* TYPHI**  **(N=80)** | **P-value Comparison** |
| --- | --- | --- | --- | --- | --- | --- | --- |
| **Azithromycin** | 83 |  | 6 |  | 77 |  | 0.0001 |
| MIC 50 |  | 3 |  | 10 |  | 3 |  |
| MIC 90 |  | 4 |  | 12 |  | 4 |  |
| Range |  | 1.5,12.0 |  | 3.0,12.0 |  | 1.5,8.0 |  |
| **Cotrimoxazole** | 82 |  | 6 |  | 76 |  | 0.001 |
| MIC 50 |  | 0 |  | 0.1 |  | 0 |  |
| MIC 90 |  | 0.1 |  | 0.2 |  | 0.1 |  |
| Range |  | 0.0,256.0 |  | 0.1,0.2 |  | 0.0,256.0 |  |
| **Amoxycillin** | 83 |  | 6 |  | 77 |  | 0.0001 |
| MIC50 |  | 0.5 |  | 0.15 |  | 0.8 |  |
| MIC90 |  | 1 |  | 1.8 |  | 1 |  |
| Range |  | 0.2,256.0 |  | 1.0,2.0 |  | 0.2,256.0 |  |
| **Chloramphenicol** | 83 |  | 6 |  | 77 |  | 0.0003 |
| MIC50 |  | 3 |  | 10 |  | 3 |  |
| MIC90 |  | 8 |  | 14 |  | 4 |  |
| Range |  | 0.2,256.0 |  | 4.0,16.0 |  | 0.2,256.0 |  |
| **Ceftriaxone** | 83 |  | 6 |  | 77 |  | 0.35 |
| MIC50 |  | 0.1 |  | 0.1 |  | 0.1 |  |
| MIC90 |  | 0.2 |  | 0.2 |  | 0.2 |  |
| Range |  | 0.0,2.0 |  | 0.1,0.2 |  | 0.0,2.0 |  |
| **Ciprofloxacin** | 83 |  | 6 |  | 77 |  | 0.22 |
| MIC50 |  | 0.2 |  | 0.2 |  | 0.2 |  |
| MIC90 |  | 0.1 |  | 0.4 |  | 0.4 |  |
| Range |  | 0.0,32.0 |  | 0.0,0.5 |  | 0.0,32.0 |  |
| **Ofloxacin** | 83 |  | 6 |  | 77 |  | 0.046 |
| MIC50 |  | 0.2 |  | 0.6 |  | 0.2 |  |
| MIC90 |  | 0.5 |  | 0.9 |  | 0.4 |  |
| Range |  | 0.0,12.0 |  | 0.1,1.0 |  | 0.0,12.0 |  |

(n/N) =number of patients

MIC=minimum inhibitory concentration. MIC 50=minimum inhibitory concentration at the 50th percentile. MIC 90=minimum inhibitory concentration at the 90th percentile.

**Supplementary Table 8:** Results of randomised control trials and observational studies using co-trimoxazole (based on Pubmed search)

| **S. no** | **Study** | **Drug Dose**  **mg/day**  **(Duration=14 days)** | **Mean FCT** | **Treatment failure** | **Relapse** |
| --- | --- | --- | --- | --- | --- |
|  | **Randomised control trials** |  |  |  |  |
| 1 | 1972  Double-blind trial with chloramphenicol and the combination trimethoprim-sulfamethoxazole in typhoid  (N=37)/75  **Reference no 16** | 800 | Not clearly mentioned.  Fever cleared by 4-5 days | 2/37=5.6% | Not mentioned |
| 2 | 1976  Comparative efficacy of chloramphenicol, ampicillin, and co-trimoxazole in the treatment of typhoid fever (N=21)/92  **Reference no 34** | 1600 | 6.9 days | 4/21=19% | No relapse  (3 weeks) |
| 3 | 1978  A Comparative Trial of Co-Trimoxazole and Chloramphenicol in Typhoid and Paratyphoid Fever  N=42/91  **Reference no 33** | 1600 | 5.0 | 4/42=9.5% | No relapses  (3 weeks) |
| 4 | 1982  Response of typhoid fever caused by chloramphenicol-susceptible and chloramphenicol-resistant strains of Salmonella typhi to treatment with trimethoprim-sulfamethoxazole  (N=33)/60  **Reference no 15** | 1600 | 5.8 days | 3/33=9% | Not mentioned |
| 5 | 1985  Effect of a new sulfa-trimethoprim combination (trimethoprim-sulfamethopyrazine) in typhoid fever  (N=35)/72  **Reference no 17** | 1600 | 5.51 days | 1/35=2% | Not mentioned |
| 6 | 1988  Prospective randomized comparative trial of perfloxacin versus co-trimoxazole in the treatment of typhoid fever in adults  (N=18)/42  **Reference no 14** | 1600 | 7.75 days | No failure | No relapse (1 month). |
| 7 | 1989  Comparative study of ciprofloxacin versus co-trimoxazole in the treatment of Salmonella enteric fever  (N=20)/40  **Reference no 18** | 1600 | Not clearly mentioned.  Most were afebrile by day 5 | 2/20=10% | Not mentioned. |
|  | Observational studies |  |  |  |  |
| 1 | 1968  Trimethoprim and sulphamethoxazole in typhoid.  (N=6)/6  <https://www.ncbi.nlm.nih.gov/pubmed/5673964> | 3200 | 6 days | No failure | Not mentioned  No follow-up |
| 2 | 1975  Co-Trimoxazole Compared to Chloramphenicol in the Treatment of Enteric Fever  <https://www.ncbi.nlm.nih.gov/pubmed/809835>  (N=60)/98 | 1600mg/day | 5.1 days | 1/17=5.8% | No Relapse  (4-6 weeks) |
| 3 | 1992  Mecillinam, mecillinam/amoxicillin and trimethoprim-sulfamethoxazole for treatment of typhoid fever in children.  <https://www.ncbi.nlm.nih.gov/pubmed/1454448>  (N=10)/30 | 24mg/kg | 5.7 days | No failures report | Didn’t report  relapse |

N= number of subjects receiving co-trimoxazole/ out of total number randomised

FCT= Fever clearance time
